# K^+^/Cl^−^ cotransporter 2 (KCC2) and Na^+^/${\text{HCO}}_{3}^{-}$ cotransporter 1 (NBCe1) interaction modulates profile of KCC2 phosphorylation

**DOI:** 10.3389/fncel.2023.1253424

**Published:** 2023-10-10

**Authors:** Abhishek Pethe, Mira Hamze, Marina Giannaki, Bernd Heimrich, Igor Medina, Anna-Maria Hartmann, Eleni Roussa

**Affiliations:** ^1^Department of Molecular Embryology, Faculty of Medicine, Institute for Anatomy and Cell Biology, Albert-Ludwigs-Universität Freiburg, Freiburg, Germany; ^2^INMED, INSERM, Aix-Marseille University, Marseille, France; ^3^Department of Neuroanatomy, Faculty of Medicine, Institute for Anatomy and Cell Biology, Albert-Ludwigs-Universität Freiburg, Freiburg, Germany; ^4^Division of Neurogenetics, Faculty VI, School of Medicine and Health Sciences, Carl von Ossietzky University Oldenburg, Oldenburg, Germany; ^5^Research Center for Neurosensory Science, Carl von Ossietzky University Oldenburg, Oldenburg, Germany

**Keywords:** GABA, potassium chloride cotransporter, sodium bicarbonate cotransporter, phosphoregulation, pH

## Abstract

K^+^/Cl^−^ cotransporter 2 (KCC2) is a major Cl^−^ extruder in mature neurons and is responsible for the establishment of low intracellular [Cl^−^], necessary for fast hyperpolarizing GABA_A_-receptor mediated synaptic inhibition. Electrogenic sodium bicarbonate cotransporter 1 (NBCe1) is a pH regulatory protein expressed in neurons and glial cells. An interactome study identified NBCe1 as a possible interaction partner of KCC2. In this study, we investigated the putative effect of KCC2/NBCe1 interaction in baseline and the stimulus-induced phosphorylation pattern and function of KCC2. Primary mouse hippocampal neuronal cultures from wildtype (WT) and *Nbce1*-deficient mice, as well as HEK-293 cells stably transfected with KCC2^WT^, were used. The results show that KCC2 and NBCe1 are interaction partners in the mouse brain. In HEK^KCC2^ cells, pharmacological inhibition of NBCs with S0859 prevented staurosporine- and 4-aminopyridine (4AP)-induced KCC2 activation. In mature cultures of hippocampal neurons, however, S0859 completely inhibited postsynaptic GABA_A_R and, thus, could not be used as a tool to investigate the role of NBCs in GABA-dependent neuronal networks. In *Nbce1*-deficient immature hippocampal neurons, baseline phosphorylation of KCC2 at S940 was downregulated, compared to WT, and exposure to staurosporine failed to reduce pKCC2 S940 and T1007. In *Nbce1*-deficient mature neurons, baseline levels of pKCC2 S940 and T1007 were upregulated compared to WT, whereas after 4AP treatment, pKCC2 S940 was downregulated, and pKCC2 T1007 was further upregulated. Functional experiments showed that the levels of GABA_A_R reversal potential, baseline intracellular [Cl^−^], Cl^−^ extrusion, and baseline intracellular pH were similar between WT and *Nbce1*-deficient neurons. Altogether, our data provide a primary description of the properties of KCC2/NBCe1 protein-protein interaction and implicate modulation of stimulus-mediated phosphorylation of KCC2 by NBCe1/KCC2 interaction—a mechanism with putative pathophysiological relevance.

## 1. Introduction

K^+^/Cl^−^ cotransporter 2 (KCC2), a product of the *Slc12a5* gene, is a major Cl^−^ extruder in mature neurons and warrants low [Cl^−^]_i_, a prerequisite for fast hyperpolarizing GABA_A_-receptor (GABA_A_R) mediated synaptic inhibition (Kaila, 1994; Rivera et al., 1999). KCC2 expression parallels overall brain maturation and is the crucial molecular player for the developmental “switch” in GABA function, from depolarizing and excitatory in immature neurons to hyperpolarizing and inhibitory functions in mature neurons (Clayton et al., 1998; Lu et al., 1999; Rivera et al., 1999; Gulyás et al., 2001; Mikawa et al., 2002; Stein et al., 2004; Ge et al., 2006). Compromised KCC2 function leads to dysregulation of Cl^−^ homeostasis and is implicated in developmental, acquired, and degenerative neurological disorders, among them epilepsy, neuropathic pain, autism spectrum disorders, schizophrenia, and following brain trauma, documented in several *in vivo* and *in vitro* studies (Payne et al., 2003; Boulenguez et al., 2010; Kahle et al., 2014; Puskarjov et al., 2014; Merner et al., 2015). Based on its pathophysiological importance, KCC2 is considered a putative potent therapeutic target.

KCC2 is a large and complex 12 transmembrane domain membrane protein harboring a large number of phosphorylatable regulatory sites (Weber et al., 2014; Zhang et al., 2020). Phosphorylation at S940, T906, and T1007 has been investigated in detail and shown to critically control KCC2's ion-transport activity and/or surface expression. Dephosphorylation of T906 and T1007 and phosphorylation of S940 activate KCC2. Phosphorylation at S932, T934, S937, T1009, and Y1087 regulates KCC2 transport as well, whereas phosphorylation at several additional sites has no impact on KCC2 activity and their role remains obscure (reviewed by Hartmann and Nothwang, 2022).

The regulatory action of KCC2 in the brain occurs via at least two distinct mechanisms implicating electroneutral K^+^/Cl^−^ ion extrusion (Payne et al., 1996) and ion-transport independent interaction with cytoskeleton-associated molecules (Li et al., 2007). More recent works have extended later observation by revealing the putative interaction of KCC2 with a plethora of molecules (Mahadevan et al., 2017; Smalley et al., 2020). The functional importance of the KCC2 interaction with some molecules has already been studied (Li et al., 2007; Ivakine et al., 2013; Llano et al., 2015; Roussa et al., 2016; Dargaei et al., 2018), whereas the importance of the interaction with the majority of interactome partners remains unknown (reviewed in detail by Virtanen et al., 2021). One of the recently discovered interactome partners of KCC2 is the electrogenic Na^+^/${\text{HCO}}_{3}^{-}$ cotransporter 1 (NBCe1) (Smalley et al., 2020).

In contrast to the more restricted expression of KCC2, NBCe1, a product of the *Slc4a4* gene, reveals a broad expression within and outside the CNS (reviewed by Parker and Boron, 2013). Mutations of the *Slc4a4* gene are accompanied by neurological signs, such as intellectual disability, ocular abnormalities, migraine, episodic ataxia, and occasionally epilepsy (Igarashi et al., 1999; Suzuki et al., 2010). In the CNS, NBCe1 is predominantly expressed in astrocytes, regulates astrocytic intracellular pH, and may modulate extracellular and synaptic pH (reviewed in Deitmer and Rose, 2010). In astrocytes, NBCe1 may operate in the inward or outward mode (Theparambil et al., 2014; Theparambil and Deitmer, 2015) and is not only the molecular component for the astrocytic depolarization-induced acidification (DIA) but also protects the brain milieu from acidification by counteracting the neuronal activity-associated transient extracellular acid load (Theparambil et al., 2020). Although neurons also express NBCe1, albeit to a lesser extent compared to astrocytes (Schmitt et al., 2000; Rickmann et al., 2007; Majumdar et al., 2008), there is limited research on the potential impact of neuronal NBCe1 under both physiological and pathological conditions. Under physiological conditions, members of the SLC4 family are involved in the regulation of intracellular pH in neurons. Among them, SLC4A8 (Chen et al., 2008; Sinning et al., 2011) and SLC4A10 (Jacobs et al., 2008) have been studied. Although SLC4A4 apparently is not a crucial molecular player in the regulation of neuronal pH homeostasis under physiological conditions, strikingly, the studies in neurons that are available so far describe SLC4A4 regulation following pathophysiological conditions, thus indicating the putative function of SLC4A4. For instance, *in vitro*, hippocampal neurons respond to sustained membrane depolarization by an acid extrusion process resembling that of astrocytes, which was attributed, at least in part, to the activation of neuronal NBCe1 (Svichar et al., 2011). Additionally, extracellular acid-base changes alter trafficking and the surface expression of NBCe1 variants in hippocampal neurons (Oehlke et al., 2013). *In vivo*, NBC immunoreactivity was upregulated in several types of hippocampal neurons after ischemia/reperfusion (Sohn et al., 2011) or seizure (Kang et al., 2002), pathologies in which KCC2 is the subject of regulation as well.

In the present study, we investigated the putative effect of KCC2/NBCe1 interaction in the stimulus-induced phosphorylation pattern and function of KCC2. In hippocampal neurons, the *Nbce1* deficiency resulted in altered KCC2 phosphorylation patterns at both baseline and following treatment with staurosporine or 4-aminopyridine (4AP), whereas E_GABA_ and Cl^−^ extrusion rates were comparable to wildtype neurons. These data implicate modulation of stimulus-mediated phosphorylation of KCC2 by NBCe1/KCC2 interaction, a mechanism with putative pathophysiological relevance.

## 2. Materials and methods

### 2.1. Antibodies and reagents/chemicals

**Figure 1:**
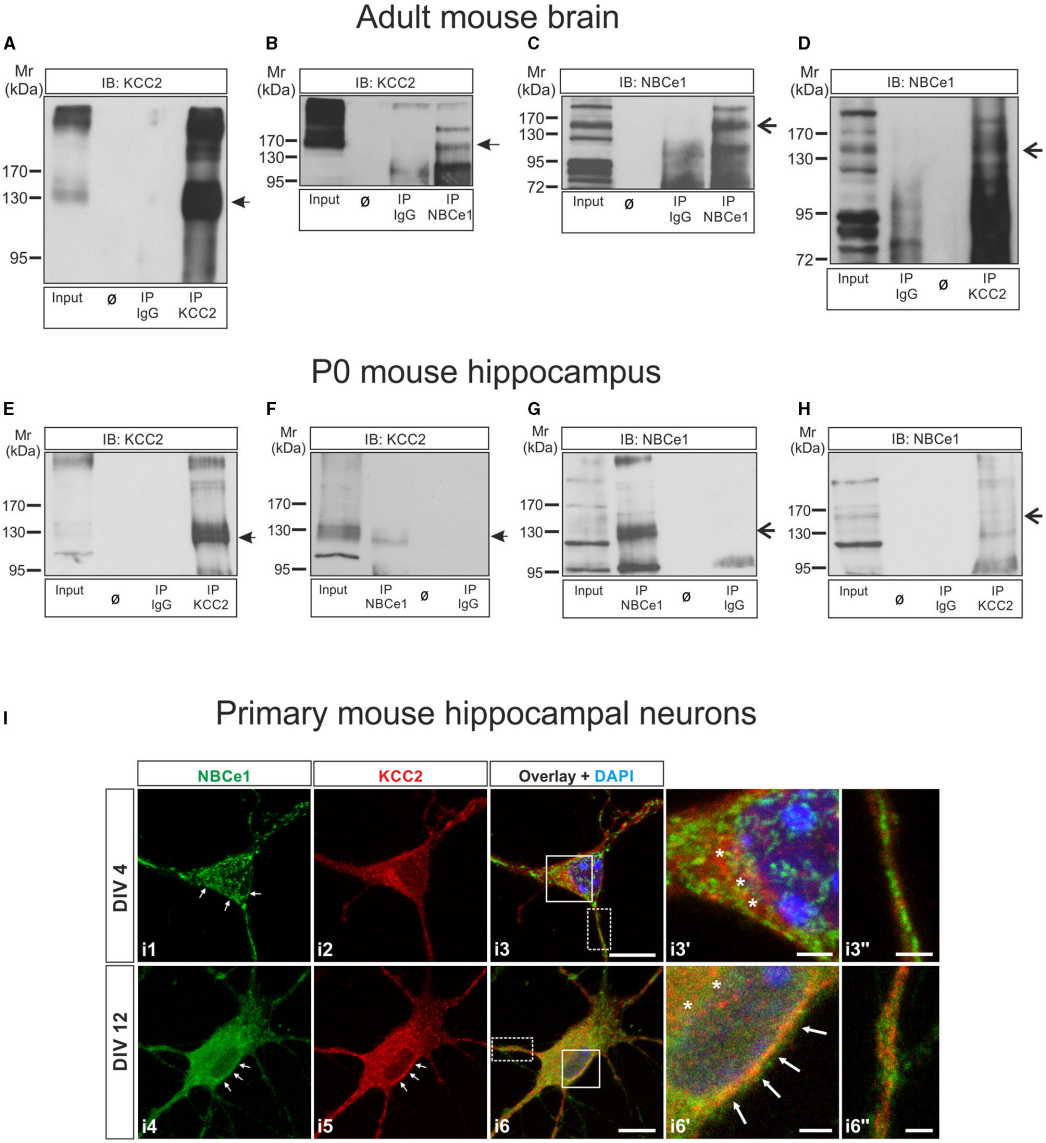
KCC2 and NBCe1 are interaction partners *in vitro* and *in vivo*. **(A–D)** Co-immunoprecipitation of KCC2 and NBCe1 in crude membranes from whole mouse brain. Enrichment of KCC2 **(A)** and NBCe1 **(C)** following immunoprecipitation (IP) of the respective proteins. Antibodies against KCC2 **(D)** and NBCe1 **(B)**, but not IgG **(B, D)**, were able to immunoprecipitate NBCe1 and KCC2, respectively, as detected by immunoblotting. **(E–H)** Co-immunoprecipitation of KCC2 and NBCe1 in homogenates from newborn (postnatal day 0; P0) mouse hippocampus. Enrichment of KCC2 **(E)** and NBCe1 **(G)** following the immunoprecipitation (IP) of the respective proteins. Antibodies against KCC2 **(H)** and NBCe1 **(F)**, but not IgG **(F, H)**, were able to immunoprecipitate NBCe1 and KCC2, respectively, as detected by immunoblotting. Ø represents empty lanes. In total, 500 μg of protein (input) or IP derived from 500 μg of protein was loaded. **(I)** Double immunofluorescence for KCC2 (red) and NBCe1 (green) on mouse primary hippocampal neurons at day *in vitro* (DIV) 4 and DIV12, using confocal microscopy, illustrates the colocalization of the proteins in the cytosol (asterisks) and/or in the plasma membrane (arrows) of the neurons. i3′ and i6′ represent higher magnification of the solid white-boxed areas in i3 and i6, respectively. i3^′′^ and i6^′′^ represent higher magnification of the dotted white-boxed areas in i3 and i6, respectively. Nuclei were labeled with DAPI (blue). Scale bar: 10 μm, and in i3′, i3^′′^, i6′, and i6^′′^: 2 μm.

The following antibodies were used as primary antibodies: anti-KCC2 rabbit polyclonal (Millipore Cat# 07-432, RRID: AB_310611), anti-KCC2 mouse monoclonal (NeuroMab Cat#N1/12, RRID: AB_2877330), anti-phosphorylated KCC2 (S940) rabbit polyclonal (Origene Cat# TA309219), normal mouse IgG (Millipore Cat# 12-371, RRID: AB_145840), anti-SLC4A4 rabbit polyclonal (Abcam Cat# ab187511 and Atlas Antibodies Cat# HPA035628 RRID: AB_2674708; Figure 1I), anti-β-actin mouse monoclonal (Proteintech Cat# 66009-1-Ig, RRID: AB_2687938), and anti-β-III-tubulin mouse monoclonal (Sigma-Aldrich Cat# T5076, RRID: AB_532291). For immunocytochemistry, anti-phosphorylated KCC3A (T1039; corresponding to KCC2A phospho-T1007) sheep polyclonal antibodies (Sheep# S961C) were obtained from the MRC Protein Phosphorylation and Ubiquitination Unit of the University of Dundee. For immunofluorescence, donkey-anti-rabbit AlexaFluor594 (Jackson ImmunoResearch Labs Cat# 711-585-152, RRID: AB_2340621) or donkey-anti-sheep AlexaFluor594 (Jackson ImmunoResearch Labs Cat# 713-585-147, RRID: AB_2340748) were used as secondary antibodies. For Western blots, horse-anti-rabbit IgG (Cell Signaling Technology Cat# 7074, RRID: AB_2099233) coupled with horseradish peroxidase was used as a secondary antibody. 4-aminopyridine (Tocris Cat# 0940), Staurosporine (Tocris Cat# 1285), and S0859 (Sigma-Aldrich Cat# SML0638) were used for treatments.

### 2.2. Animals

All protocols were carried out in accordance with German ethical guidelines for laboratory animals and approved by the Institutional Animal Care and Use Committee of the University of Freiburg and the City of Freiburg (authorizations: X19/09C, X22/11B, and G-21-140). Adult C57BL/6N mice (strain code 027) of either sex were maintained on a 12 h dark/light cycle with food and water *ad libitum*. Mice were sacrificed by cervical dislocation, and all efforts were made to minimize suffering. *Slc4a4* deficient mice have been described earlier (Gawenis et al., 2007).

### 2.3. Tl^+^ based KCC2 flux measurements in HEK-293 cells

Transport activity of KCC2 was determined by Cl^−^-dependent uptake of Tl^+^ in HEK-293 cells as described previously (Hartmann et al., 2010, 2021). To initiate the flux measurement, the medium in the 96-well culture dish was replaced by 80 μL hypotonic preincubation buffer (100 mM N-methyl-D-glucamine-chloride, 5 mM Hepes, 5 mM KCl, 2 mM CaCl_2_, 0.8 mM MgSO_4_, 5 mM glucose, pH 7.4; osmolarity: 175 mmol/kg ± 2) with 2 μM FluoZin-2 AM dye (Invitrogen) plus 0.2% (wt/vol) pluronic F-127 (Invitrogen) and incubated for 48 min at room temperature. Afterward, the cells were washed three times with 80 μL preincubation buffer and incubated for 15 min with 80 μL preincubation buffer, including 0.1 mM ouabain, to block the activity of the Na^+^/K^+^ ATPase. Then, the 96-well plate was placed into a fluorometer (Fluoroskan FL, Thermo Scientific), and each well was injected with 40 μL 5× thallium stimulation buffer (12 mM Tl_2_SO_4_, 100 mM N-methyl-D-glucamine 5 mM Hepes, 2 mM KCl, 2 mM CaCl_2_, 0.8 mM MgSO_4_, and 5 mM glucose, pH 7.4). The fluorescence was measured in a kinetic-dependent manner (excitation 485 nm, emission 538 nm, 1 frame in 6 s in a 200 s period) across the entire cell population in a single well. The transport activity was calculated using a linear regression of the initial values of the slope of Tl^+^- stimulated fluorescence increase.

To investigate the effect of staurosporine, 4AP, and NBC inhibitor S0589 on KCC2 activity, 50 μM of S0859 was applied to the preincubation buffer 30 min prior to flux measurement. Then, 10 μM of staurosporine was applied to the preincubation buffer for 15 min, or 100 μM of 4AP was applied to the preincubation buffer 60 min prior to flux measurement.

### 2.4. Primary cultures of mouse E17.5 hippocampal neurons

Hippocampal neurons were isolated from either C57BL/6N, wildtype, or *Slc4a4* deficient mice at embryonic day 17.5 (E17.5) of gestation, as described earlier (Roussa et al., 2016). At day *in vitro* (DIV) 4, cultures were treated with 10 μM staurosporine for 15 min or at DIV 12 with 4AP (100 μM) for 60 min and subsequently processed for immunocytochemistry, intracellular H^+^, and Cl^−^ measurements or electrophysiological recordings.

To effectively distinguish between neurons and astrocytes, we employed the following morphological criteria: size and shape of the nucleus, the nucleus-to-soma size ratio, as well as shape, length, and orientation of the cellular processes. Hippocampal neurons were mainly identified based on their somatic architecture, characterized by extended dendrite-like processes. Any surviving astrocytes were readily distinguishable through their distinctive protoplasmic morphology or by the presence of multiple, often more numerous than observed in neurons, shorter processes reminiscent of filopodia.

### 2.5. Preparation of crude membranes

Mouse adult brain tissue was suspended in a suspension buffer containing 250 mM sucrose, 40 mM MOPS, 0.1 mM EGTA, and 0.1 mM MgSO_4_ (pH adjusted to 7.4) and homogenized using a homogenizer (IKA-RW15) at 11,000 rpm with at least 20 up-and-down motions, on ice. The homogenate was centrifuged at 1,500 × g at 4°C for 10 min, and the supernatant was collected and centrifuged at 1,000 × g at 4°C for 10 min. The supernatant thus obtained was centrifuged at 4,000 × g at 4°C for 10 min. The pellet was discarded, and the supernatant was centrifuged in an ultracentrifuge (Beckmann-Coulter XPN90, SW-41 Ti rotor) at 35,000 × g for 30 min to pellet down the crude membrane fractions. The pellet was then resuspended in non-denaturing lysis buffer (IP buffer; 50 mM Tris-HCl pH 7.4, 300 mM NaCl, 5 mM EDTA, and 1%Triton X-100) and processed further.

### 2.6. Immunoprecipitation

Whole tissue homogenate from P0 mouse hippocampus and crude membranes obtained from adult mouse whole brain were suspended in 1 mL of non-denaturing lysis buffer. Protein concentration was determined using a Thermo Fisher Scientific NanoDrop 2000 spectrophotometer (at absorbance 280 nm). Immunoprecipitation was essentially performed as previously described (Roussa et al., 2016) with minor modifications. Then, 500 μg of protein was mixed with 75 μl protein A-Sepharose 4B conjugate beads (Invitrogen, Cat# 10-1042) 1:1 in immunoprecipitation buffer, i.e., non-denaturing lysis buffer containing 0.1% triton X-100 and incubated overnight at 4°C on an end-to-end roller. After centrifugation for 5 min at 2,300 × g, the precleared cell lysate was added to protein A-Sepharose beads conjugated with antibody and incubated overnight at 4°C on an end-to-end roller. To prepare antibody-conjugated beads, protein A-Sepharose 4B conjugate beads were blocked with 10% bovine serum albumin (BSA) in immunoprecipitation buffer and then incubated overnight at 4°C on an end-to-end roller with 5 μg of antibody (anti-KCC2 or anti-SLC4A4 or mouse IgG) in immunoprecipitation buffer. The beads were centrifuged for 5 min at 2,300 × g, and the precleared cell lysate was added as described above. Subsequently, the beads were centrifuged and washed multiple times in an immunoprecipitation buffer, and the supernatant was discarded. Bound proteins and their interaction partners were resuspended in 30 μL 3× sample buffer [62.5 mM Tris-HCl pH 6.8, 2% sodium dodecyl sulfate (SDS), 10% glycine, 5% β-mercaptoethanol, and 0.001% Bromophenol Blue], heated for 5 min at 95°C and immediately cooled down on ice, and processed by SDS-PAGE.

### 2.7. Immunoblotting

Protein concentration was determined by a Thermo Scientific NanoDrop 2000 spectrophotometer (at absorbance 280 nm). Electrophoresis and blotting procedures were performed as described (Khakipoor et al., 2017). Primary antibodies were diluted as follows: KCC2 1:5,000–1:10,000, SLC4A4 1:5,000, β-actin 1:30,000, and β-III-tubulin 1:20,000. Blots were developed in enhanced chemiluminescence reagents (Signalfire ECL reagent, Cell Signaling Cat# 12630S), and signals were visualized on X-ray films (Amersham Hyperfilm ECL, Cytiva Cat# 28906837).

### 2.8. Immunocytochemistry

HEK-293 cells grown on poly-L-lysine-coated coverslips were fixated for 10 min with 4% paraformaldehyde (PFA) in 0.2 M phosphate buffer. Afterward, the cells were washed three times with phosphate buffer saline (PBS) before the blocking solution (2% BSA and 10% goat serum in PBS) was applied for 30 min. Primary antibody solution (anti-KCC2 N1-12; 1:1,000; Neuromab) was added to the carrier solution (0.3% Triton X-100, 1% BSA, and 1% goat serum in PBS) and incubated for 1 h. After washing three times with PBS, the secondary antibody, which was conjugated to a fluorescent probe (Alexa Flour 488 goat anti-mouse; 1:1,000; Thermo Fisher Scientific, Bremen, Germany), was added to the carrier solution and incubated for 1 h. Again, the cells were washed three times with PBS and completely dried. The dried coverslips were mounted onto glass slides with Mowiol (Roth) and 4′,6-diamidine-2-phenylindole (DAPI, 1:1,000; Roth). Photomicrographs were taken using an Olympus fluorescence microscope (Olympus BX63).

Primary hippocampal neurons were fixed with 4% PFA for 30 min at room temperature. The cells were washed with PBS and treated with 1% SDS for 5 min, blocked with 1% BSA and incubated with primary antibody α-KCC2 (1:300), α-pKCC2 S940 (1:300), or α-pKCC3 T1039/pKCC2 T1007 (5 μg/mL; with 100 μg/mL of the non-phosphorylated form of the phosphorylated peptide used to raise the antibody), and anti-NBCe1 (1:300) in 1%BSA/PBS overnight at 4°C. The cells were washed with PBS and incubated with goat anti-rabbit IgG or goat anti-sheep IgG coupled with AlexaFluor594 (1:400) for 1 h. Coverslips were washed with PBS and mounted with Fluoromount-G, containing DAPI (SouthernBiotech #0100-20) for nuclear staining.

For fluorescence images shown in Figure 1I, FlexAble CoraLite^®^ Plus 488 (Proteintech Cat# KFA001) and FlexAble CoraLite^®^ Plus 555 (Proteintech, Cat# KFA002) kits were used to label 0.5 μg anti-SLC4A4 rabbit polyclonal and anti-KCC2 rabbit polyclonal primary antibodies, respectively, according to the manufacturer's instructions. Cells washed with PBS, permeabilized with 1% SDS for 5 min, and blocked with 1% BSA were incubated with the primary labeled antibodies overnight at 4°C in 1% BSA/PBS. Coverslips were washed with PBS and mounted as described above.

### 2.9. Image acquisition and analysis

Images were acquired with a Leica TCS SP8 confocal microscope using an HC PL APO CS2 40×/1.30 or 63×/1.40 oil objective lens, and immunofluorescence intensity was analyzed as described previously (Chleilat et al., 2020). Within each experiment, the confocal microscope settings (laser power, detector gain, and amplifier offset) were kept the same for all scans in which the immunofluorescence intensity was compared. Z-stacks of 16–30 optical sections with a step size of 0.4 μm were taken for at least 3 separate fields of view for each experimental condition. Maximum intensity projections were created from the z-stacks. To quantify the protein expression, LAS X software was used to select the area of interest (neuronal soma) and measure the average fluorescence intensity within this area for each cell. Background subtraction was applied to the images.

For determining membrane KCC2 localization, a line was drawn in the LAS X software using the “Draw Line” tool across the neuronal cell body and through the nucleus, spanning the periphery of the cell. The line profile tool was used to examine the fluorescence distribution across the line. Cells that displayed fluorescence intensity peaks at the periphery (suggesting KCC2 expression on the cell membrane) were classified as cells with membrane expression; cells that did not exhibit these peaks were defined as cells without membrane expression. Cells expressing membrane expression were illustrated as a percentage against the total number of cells analyzed with line scans.

### 2.10. Electrophysiological recording

The gramicidin perforated whole-cell patch-clamp recordings from hippocampal neurons for WT and *Nbce1* deficient mice were performed at 23–24°C according to protocols described previously (Friedel et al., 2015; Dumon et al., 2018). Briefly, the external solution was an artificial cerebrospinal fluid (ACSF) with the following composition (in mM): 126 NaCl, 3.5 KCl, 2 CaCl_2_, 1.3 MgCl_2_, 1.2 NaH_2_PO_4_, 25 NaHCO_3_, and 11 glucose, pH 7.4 equilibrated with 95% O_2_ and 5% CO_2_. Then, 10 μM of bumetanide was added routinely to block Cl^−^ influx mediated by NKCC1—a Na^+^/K^+^/2Cl^−^ cotransporter 1. Coverslips with cultured neuronal cells were placed onto an inverted microscope and perfused with a fast perfusion system placed in front of the recording neuron to ensure effective neuron perfusion with oxygenated (95% O_2_ and 5% CO_2_) ACSF and removal of trace amounts of gramicidin that could diffuse from the patch pipette. For recording, pyramidal cells were selected based on their morphology and ability to generate spikes after establishing a perforated patch configuration. Patch pipettes (5 megaohms) were filled with a solution containing 150 mM KCl, 10 mM Hepes, and 20 μg/ml gramicidin A (pH 7.2). Giga seals were formed by rapidly approaching the patch pipette to the neuronal surface without applying positive pressure for 5–10 s (to diminish the leak of gramicidin). After sealing, series resistance (Rs), membrane resistance (Rm), and neuron capacitance (C) were monitored routinely at a Vh of −80 mV with 5 mV hyperpolarizing pulses, typically taking 10–15 min for the series resistance to stabilize at 15–60 megaohms. Data were low pass filtered at 2 kHz and acquired at 10 kHz. Isoguvacine (30 μM) was focally applied (50–150 ms, 10,000–30,000 Pa) to the neuron soma and proximal dendrites through a micropipette connected to a Picospritzer (General Valve Corporation). Isoguvacine responses were recorded at voltages −120, −100, −80, and −60 mV. A linear regression was used to calculate the best-fit line of the voltage dependence of the synaptic currents. While recording isoguvacine responses, 1 μM TTX was included in the ACSF to block neuron spiking activity.

### 2.11. [Cl^−^]_i_ recording using Clo-pHensorN fluorescent probe

To measure the intracellular Cl^−^ changes in primary DIV12-13 neurons from WT and *Nbce1*-KO (knock-out) mice treated (and not treated) with 100 μM 4AP for 1 h, we used a ratiometric Cl^−^-sensitive probe called Clo-pHensorN (Arosio et al., 2010; Raimondo et al., 2013). At DIV 7, the neurons growing on coverslips in 12 mm well-plates were transfected by adding per well a mixture of 60 μL of Opti-MEM medium, 1.4 μL of Lipofectamine 2000 (Invitrogen), 0.3 μL of Magnetofection CombiMag (OZ Biosciences), and 0.3 μg of cDNAs encoding ClopHensorN. The mixture was incubated for 15 min at room temperature and thereafter distributed dropwise above the neuronal culture. Culture dishes were placed on a magnetic plate (OZ Biosciences) and incubated for 1–2 h at 37°C with 5% CO_2_. Transfection was terminated by the substitution of 50% of the incubation solution with a fresh culture medium.

The acquisition of fluorescence images was performed using a customized imaging setup (described previously in detail, Hartmann et al., 2017) and consecutive cell imaging using the following pairs of excitation (ex.) and emission (em.) filters: ex. 436/20; em. 520/40 (chloride sensitive channel), ex. 577/25; em. 641/75 (chloride and pH insensitive).

Images were obtained every 10 s (0.1 Hz) with a 40× objective. The duration of excitation was selected for each set of cultures to avoid obtaining the overexposed (saturated) or underexposed images. Individual coverslips were transferred to a specially designed recording chamber where they were fully submerged and superfused with oxygenated ACSF complemented with 10 μM of bumetanide at room temperature at a rate of 2–3 mL/min. For recording, pyramidal-like neurons were selected based on their morphology. The applications of ACSF containing 50 mM KCl instead of an equivalent amount of NaCl + isoguvacine (10 μM) + bumetanide (10 μM) were performed with a perfusion system. The recovery of fluorescence after Cl^−^ overload produced by KCl + isoguvacine was recorded in ACSF. The offline creation and analysis of the ratiometric images (F577/F436) was performed using Metamorph software (Roper Scientific SAS, Evry, France) as described previously (Hartmann et al., 2017). In the present study, we analyzed two parameters. The first was the mean steady-state value of F577/F436 ratio (R_577/436_) measured during 3 min and reflecting resting [Cl^−^]_i_. The second parameter was the speed of the recovery of (R_577/436_) after neuron overload with Cl^−^ achieved by 2 min application of ACSF containing 50 mM KCl instead of an equivalent amount of NaCl. The recovery speed of (R_577/436_) reflects the kinetic of Cl^−^ extrusion that is primarily KCC2-dependent (Hartmann et al., 2017).

### 2.12. Intracellular H^+^ recordings with BCECF-AM

To measure the intracellular pH changes in primary DIV12-13 neurons from WT and *Nbce1*^−/−^ mice, we used an imaging system and acetoxymethyl ester of a proton-sensitive dye, 2′,7′-bis-(carboxyethyl)-5-(and-6)-carboxyfluorescein (BCECF-AM; Invitrogen, Cat# B8806). The dye was loaded into the cells by incubating them with 2 μM BCECF-AM in ACSF-buffered saline solution for 20 min at room temperature. The cells were then mounted on a chamber of the Nikon ECLIPSE TE200 microscope and perfused continuously at room temperature with ACSF-buffered (in mM) NaCl 126, KCl 3.5, α-d-glucose 11, NaH_2_PO_4_ 1.2, NaHCO_3_ 25, MgCl_2_ 1.3, and CaCl_2_ 2, pH 7.4 in the presence or absence of 100 μM 4AP. BCECF was excited consecutively at 488 nm (proton-sensitive wavelength) and 440 nm (close to isosbestic point), and the changes in fluorescence emission were monitored at >505 nm. Images were obtained every 5 s (0.2 Hz) with a 20× water immersion objective. The fluorescence emission intensity of 488 nm excitation changes inversely with a change in [H^+^]_i_, whereas the fluorescence emission intensity of 440 nm excitation is largely pH insensitive. The changes in [H^+^]_i_ were monitored using the ratio F440/F488 (R_440/488_). The ratio was converted into pH and absolute intracellular proton concentrations ([H^+^]_i_) using the nigericin-based (4 μM) calibration technique (Khakipoor et al., 2017, 2019). The cells were perfused with calibration solution containing the following (in mM): KCl 145, NaH_2_PO_4_ 0.4, Na_2_HPO_4_ 1.6, glucose 5, MgCl_2_ 6H_2_O 1, and Ca-DiGluconat H_2_O 1.3, adjusted at pH 6.5, 7.0, 7.5, and 8.0. To determine the baseline pH_i_, only the first 3 min from the recording were used. To calculate **Δ**pH_i_, the mean pH_i_ of wildtype (WT) controls was used as a reference and was subtracted from baseline pH_i_ from all experimental conditions (WT-ctrl, WT-4AP, KO-ctrl, and KO-4AP).

### 2.13. Statistical analysis

Statistical tests were performed as indicated in the Supplementary Tables S1–S11. All tests were performed in GraphPad Prism, Version 7.04 for Windows. Data were tested for normal distribution using a Shapiro-Wilk test and subsequently assessed for homogeneity of variance. If the data passed both tests and for comparisons between more than two groups, a one-way ANOVA and Bonferroni *post-hoc* were applied. In the figures, the normally distributed data are represented as mean and SD, where SD is a standard deviation reflecting data variability. The data that did not pass the normal distribution and equal variance tests were analyzed using the Mann-Whitney or Kruskal-Wallis non-parametric tests applied for two or multiple groups, respectively. The data showing non-normal distribution were illustrated as individual points and box plots if the sample size was <20. For sample sizes of more than 20, only boxplots were shown. For the boxplots, the box extended from the first (Q1) to the third (Q3) quartiles. The line inside the box represented the median. The whiskers defined the minimum and maximum values. For all statistical tests, *p* < 0.05 was considered statistically significant.

## 3. Results

### 3.1. KCC2 and NBCe1 colocalize and are interaction partners in the mouse brain

A recent interactome study has identified the electrogenic Na^+^/${\text{HCO}}_{3}^{-}$ cotransporter 1 (NBCe1) as a putative interaction partner of KCC2 (Smalley et al., 2020). To pursue this observation, we first performed co-immunoprecipitation experiments.

Co-immunoprecipitation of the proteins in 3–5 month-old mouse brain crude membrane preparations is illustrated in Figures 1A–D. Immunoblotting with antibodies against KCC2 revealed an immunoreactive band at ~140 kDa, whereas multiple bands were obtained after the anti-NBCe1 antibody, as previously described (Khakipoor et al., 2017). To identify the specific NBCe1 band(s), we performed immunoblot analysis on hippocampal organotypic slices derived from P2 to P4 wildtype (*Nbce1*^+/+^) and *Nbce1* deficient mice (*Nbce1*^−/−^, KO*)* that were cultured for 35 days *in vitro*. As shown in Supplementary Figure 1A in the *Nbce1*^−/−^, the ~130–140 kDa immunoreactive band (arrow) was abolished, compared to the wildtype. We further complemented this result by immunofluorescence in primary mouse DIV4 hippocampal neurons from WT and *Nbce1* deficient mice. As shown in Supplementary Figure 1B, NBCe1 immunofluorescence was greatly depleted in the KO neurons compared to WT. We, therefore, considered the ~130–140 kDa band as NBCe1 specific.

Figure 1A shows KCC2 protein at ~140 kDa highly enriched following immunoprecipitation of KCC2 (IP KCC2; Figure 1A, lane 4, arrow), compared to the input (Figure 1A, lane 1). Figure 1B shows that KCC2 could be pulled down by anti-NBCe1 antibody (IP NBCe1, lane 4, arrow) but not by IgG (IP IgG; lane 3). NBCe1 was also enriched following immunoprecipitation using an anti-NBCe1 antibody (IP NBCe1; Figure 1C, lane 4, arrow)—a result not observed in immunoprecipitation with IgG (IP IgG; Figure 1C, lane 3). NBCe1 was immunoprecipitated with KCC2 antibody (IP KCC2, Figure 1D, lane 4, arrow) but not with control IgG antibody (Figure 1D, lane 2).

To verify whether KCC2 associates with NBCe1 at earlier developmental stages as well, co-immunoprecipitation was additionally performed in newborn (postnatal day 0, P0) mouse hippocampus homogenates (Figures 1E–H) and showed similar results as obtained in the adult brain. KCC2 and NBCe1 proteins were enriched following IP with their respective antibodies (Figure 1E, IP KCC2, lane 4, arrow; Figure 1G, IP NBCe1, lane 2, arrow). Co-IP with IgG was never able to pull down either KCC2 (Figure 1E, IP IgG, lane 3) or NBCe1 (Figure 1G, IP IgG, lane 4). Again, we were able to co-immunoprecipitate KCC2 with an anti-NBCe1 antibody (Figure 1F, IP NBCe1, lane 2, arrow) as well as NBCe1 with anti-KCC2 antibody (Figure 1H, IP KCC2, lane 4, arrow).

As a next step, we examined the colocalization of NBCe1 with KCC2 in primary hippocampal neurons during neuronal maturation, namely in immature neurons at DIV4 and in differentiated neurons at DIV12 by double immunofluorescence. As shown in Figure 1I, in immature hippocampal neurons (Figure 1I, i1–i3^′′^), KCC2 was exclusively intracellularly distributed, where it colocalized with NBCe1 (asterisks, Figure 1I, i3′). At this developmental stage, NBCe1 was additionally abundant in the plasma membrane (arrows in Figure 1I, i1) and in neuronal processes (Figure 1I, i3^′′^). Colocalization of KCC2 with NBCe1 persisted in differentiated hippocampal neurons as well (Figure 1I, i4–i6^′′^). In DIV12 hippocampal neurons, the KCC2 was located in the periphery of the neuron (presumably, plasma membrane; arrows in Figure 1I, i5) and intracellular structures. The NBCe1 colocalized with KCC2 in the cytosol (asterisks in Figure 1I, i6′) and in the neuron periphery (arrows in Figure 1I, i6′). Moreover, in neuronal processes, KCC2 and NBCe1 revealed combined co- or complementary (Figure 1I, i6^′′^) localization.

Taken together, these data implicate the presence of an NBCe1/KCC2 complex *in vivo*.

### 3.2. NBC regulates staurosporine-mediated KCC2 activation

**Figure 2:**
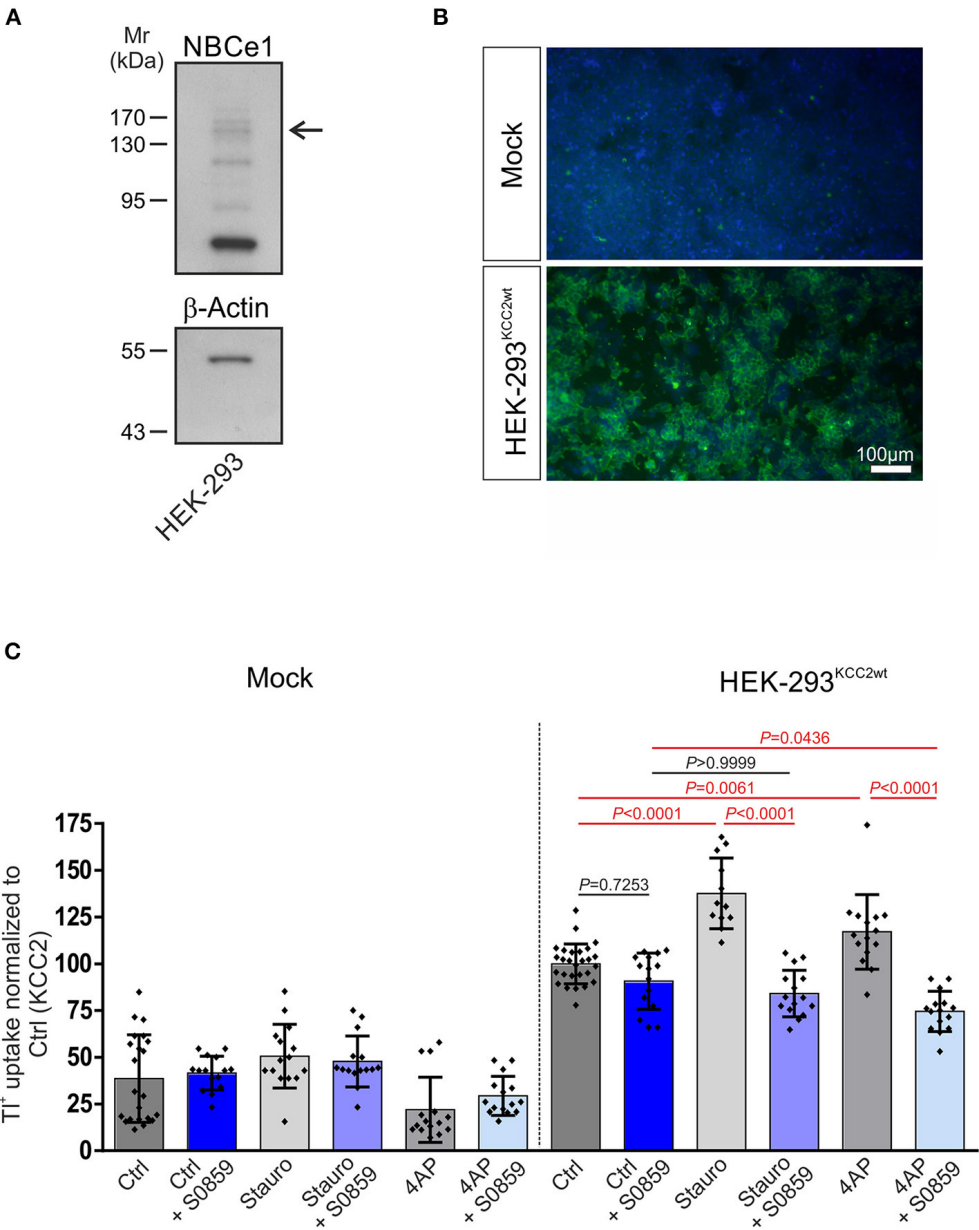
Involvement of Na^+^/${\text{HCO}}_{3}^{-}$ cotransport (NBC) in staurosporine- and 4-aminopyridine (4AP)-mediated KCC2 activation. **(A)** Immunoblot analysis in whole cell homogenates from HEK-293 cell line using NBCe1 antibody. The arrow points to the specific 130–140 kDa NBCe1 immunoreactive band. In total, 20 μg of protein was loaded. **(B)** Immunofluorescence for KCC2 (green) was detected in stably transfected KCC2^WT^ but not in mock-transfected HEK-293. **(C)** Transport activity of KCC2 in mock- or stably transfected KCC2^WT^ HEK-293 cells and following treatment with either 10 μM staurosporine or with 100 μM 4AP in the presence or absence of 50 μM NBC inhibitor S0859. Transport activity was determined by performing Tl^+^ flux measurements. The staurosporine- or 4AP-dependent significant increase of KCC2 transport activity was abolished after the application of NBC inhibitor (differences were tested for statistical significance using the one-way ANOVA and Bonferroni *post-hoc* test). The graph (mean ± SD) represents the data of three (for 4AP) to four (for staurosporine) independent measurements (each consisting of three technical replicates) normalized to KCC2^WT^. Statistically significant differences are highlighted in red.

To examine whether NBC and KCC2 coexisted in a functional complex and determine whether NBCs may affect KCC2 function, we further assayed for KCC2 function following inhibition of all NBCs via S0859 (Ch'En et al., 2008) under resting conditions and following either staurosporine-mediated KCC2 activation or exposure to the K^+^ channel blocker 4AP in HEK-293 cells. We first examined whether HEK-293 cells endogenously express NBCe1. As shown in Figure 2A, immunoblot analysis on whole cell homogenates using the specific anti-NBCe1 antibody revealed multiple immunoreactive bands, among them an immunoreactive band at ~130–140 kDa (arrow), corresponding to the molecular mass of full-length protein. Subsequently, mock-transfected HEK-293 cells or the stably transfected KCC2^WT^ HEK-293 cell line were used, and transport activity was determined by performing Tl^+^ flux measurements, as previously described (Hartmann et al., 2010, 2021). The cells were treated with either 10 μM staurosporine for 15 min or 100 μM 4AP for 60 min in the presence or absence of 50 μM S0859. As shown in Figure 2B, in mock-transfected HEK-293 cells, KCC2 immunoreactivity was absent, whereas stably transfected KCC2^WT^- HEK-293 cells revealed KCC2 immunoreactivity uniformly throughout the cells, and only the nucleus was spared. KCC2^WT^ displayed more than two-fold increased transport activity compared with mock-transfected cells (Figure 2C; Supplementary Table S1). Moreover, in mock-transfected cells, staurosporine or 4AP had no effect on Tl^+^ flux. Treatment of KCC2^WT^ HEK-293 cells with staurosporine, however, resulted in significantly increased transport activity, confirming previous observations (Weber et al., 2014; Cordshagen et al., 2018; Zhang et al., 2020)—an effect that was prevented in the presence of S0859. A similar regulation pattern was also observed when 4AP was used as a stimulus. In stable KCC2^WT^ HEK-293 cells, the application of 4AP resulted in a significant increase in KCC2 activity compared to the untreated controls, which was abolished in the presence of S0859. In contrast, S0859 had no impact on Tl^+^ uptake in control untreated KCC2^WT^ HEK-293 cells (Figure 2C; Supplementary Table S1).

The next evident step was the study of the importance of the KCC2-NBC interaction for the functioning of KCC2 in cultured hippocampal neurons. The best methodologically developed approaches to the assay of KCC2 activity in neuronal cells included whole-cell recording of the soma-dendrite gradients of GABA_A_ subtype of GABA receptor (GABA_A_R) currents and gramicidin-perforated patch clamp recordings of reversal potential of GABA_A_R (E_GABA_) (reviewed in detail by Medina and Pisella, 2020). Therefore, our first envisaged tests of the KCC2 activity included recordings of GABA_A_R responses from cultured hippocampal neurons during the application of 50 μM of S0859, an inhibitor of NBCs.

Surprisingly, our experiments revealed that the application of 50 μM of S0859 provoked rapid (onset time 2–4 min) and complete inhibition of spontaneous GABA_A_R-mediated currents (Supplementary Figure 2), an effect that presumably was not related to the inhibition of NBCs. No inhibitory effect was observed on glutamate-mediated postsynaptic currents. The S0859 acted, supposedly, by inhibiting postsynaptic GABA_A_R responses as it also strongly abolished the responses induced by focal applications of 30 μM isoguvacine, a selective GABA_A_R agonist (Supplementary Figure 2).

Taken together, these results show that S0859 acts as a potent inhibitor of GABA_A_R responses and, therefore, cannot be used to study the functional properties of GABA-dependent neuronal networks.

### 3.3. NBCe1 impairs stimulus-induced phosphorylation of KCC2 at S940 and T1007 in immature and differentiated neurons

**Figure 3:**
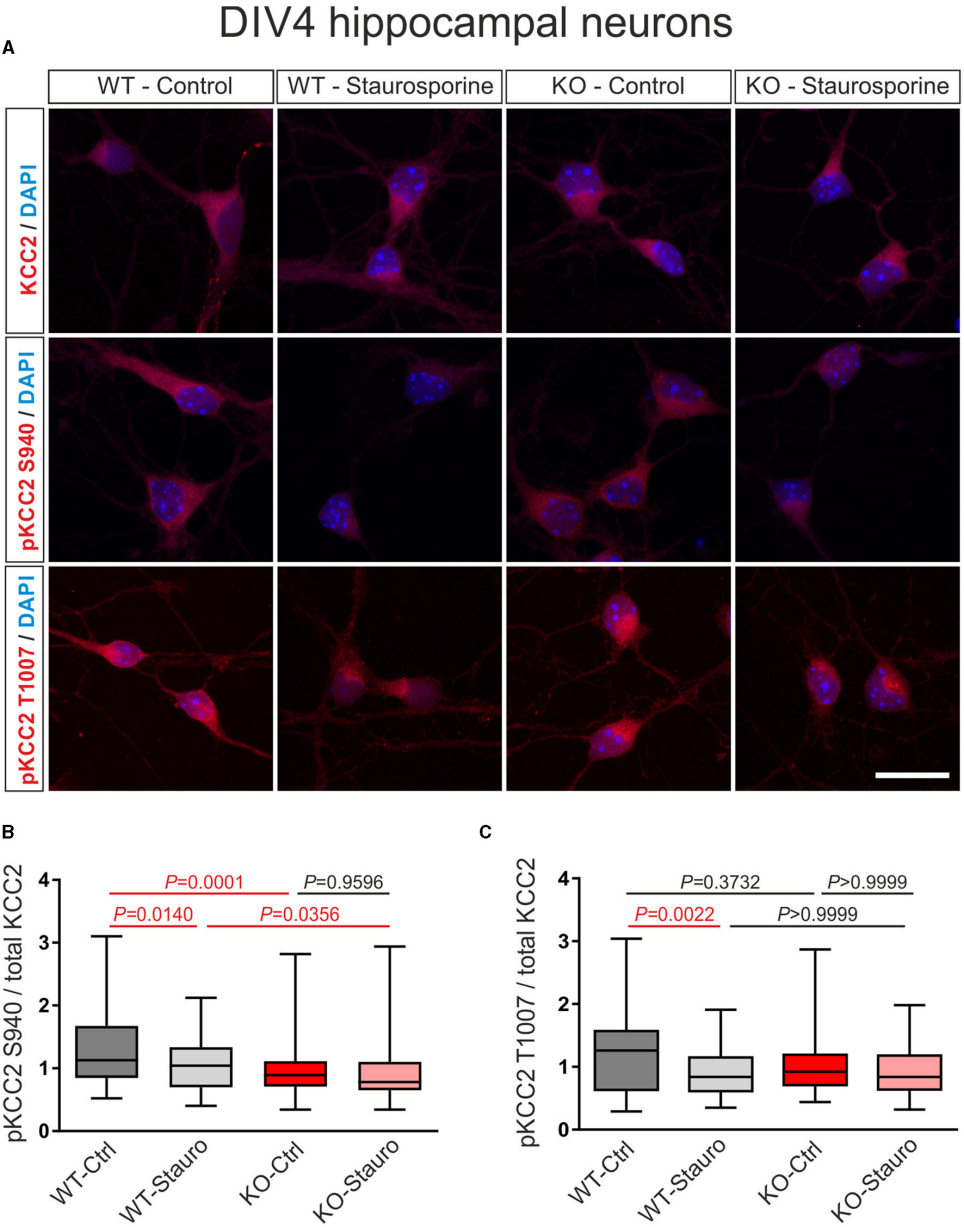
Stauroporine-induced KCC2 phosphorylation state in immature DIV4 hippocampal neurons from wildtype (WT) and *Nbce1* deficient (KO) mice. **(A)** Immunofluorescence confocal microscopy for KCC2, pKCC2 at S940, and pKCC2 at T1007 (all represented in red) on mouse primary immature hippocampal neurons for controls (Ctrl) and after exposure to 10 μM staurosporine (Stauro) for 15 min. Nuclei were labeled with DAPI (blue). Scale bar: 20 μm. **(B, C)** Boxplots showing quantification of the ratio of immunofluorescence intensities in WT and KO after analyzing a total of 75–140 cells for each experimental condition from 3 to 4 animals per genotype. **(B)** The baseline pKCC2 S940/total KCC2 ratio was downregulated in KO, compared to WT, and the staurosporine failed to reduce pKCC2 S940/total KCC2 in KO. **(C)** Exposure to staurosporine downregulated pKCC2 T1007/total KCC2 ratio in WT but not in KO. Differences were tested for statistical significance using the Kruskal-Wallis and Dunn *post-hoc* test. Statistically significant differences are highlighted in red.

**Figure 4:**
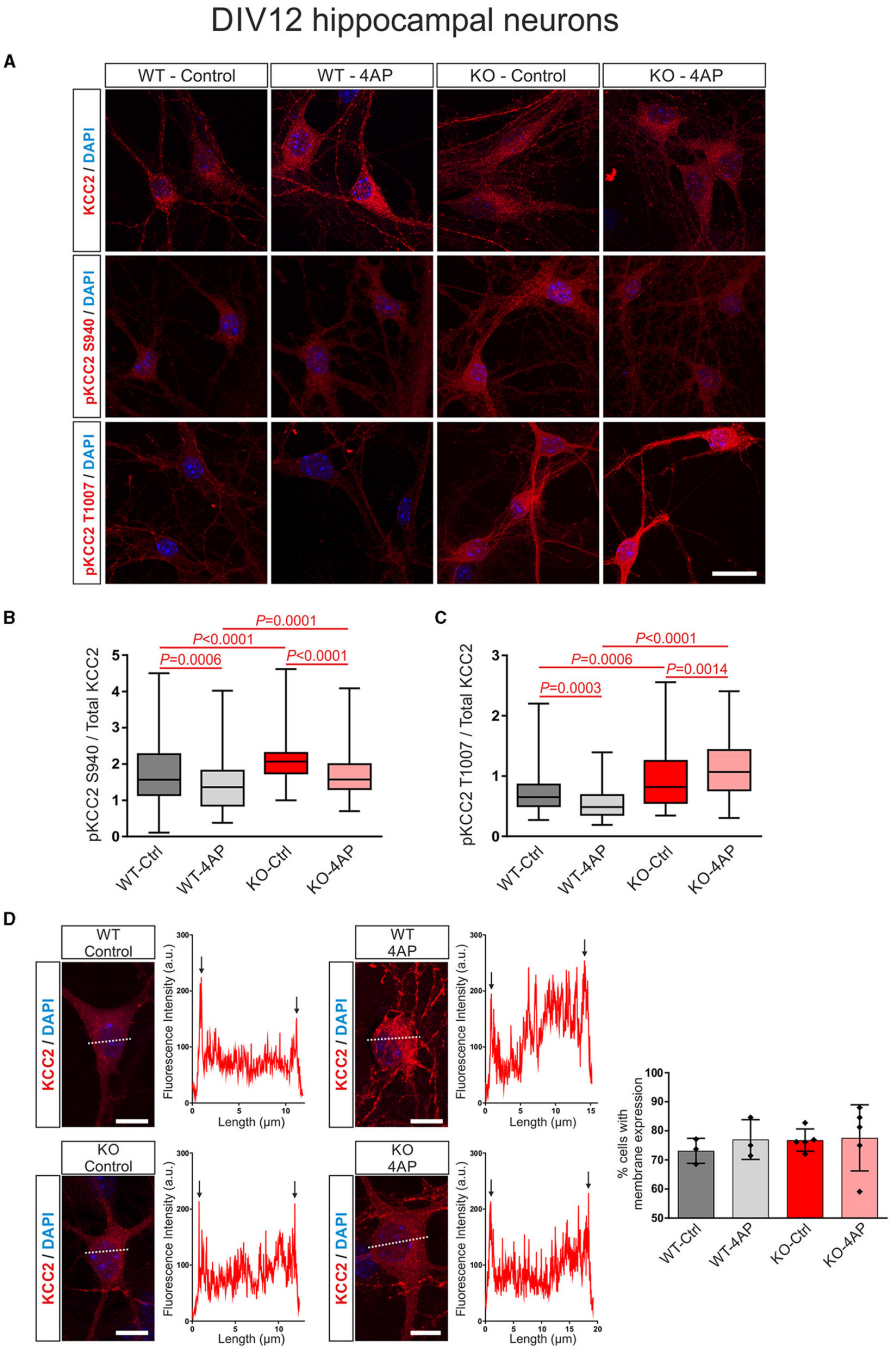
4AP-induced KCC2 phosphorylation and membrane expression in differentiated DIV12 hippocampal neurons from wildtype (WT) and *Nbce1* deficient (KO) mice. **(A)** Immunofluorescence confocal microscopy for KCC2, pKCC2 at S940, and pKCC2 at T1007 (all represented in red) on mouse primary DIV12 hippocampal neurons for WT and KO. Nuclei were labeled with DAPI (blue). **(B, C)** Boxplots showing the quantification of the ratio of immunofluorescence intensities in WT and KO derived from analyzing 60–160 cells for each experimental condition from 3 to 5 animals per genotype. **(B)** The ratio of pKCC2 S940/total KCC2 was upregulated in the KO at baseline, compared to the WT. The 4AP treatment downregulated the ratio of pKCC2 S940/total KCC2 in both WT and KO. **(C)** The ratio of pKCC2 T1007/total KCC2 was upregulated in baseline in KO. The 4AP upregulated the ratio of pKCC2 T1007/total KCC2, compared to untreated cells in KO, but reduced it in WT neurons. Differences were tested for statistical significance using the Kruskal-Wallis and Dunn *post-hoc* test. **(D)** Representative line scans following immunofluorescence for KCC2 (red) and quantification of neurons exhibiting membrane KCC2 (in %) showed no difference between WT and KO. Arrows point to spikes of fluorescence intensity at the periphery of the cell. A total of 50–140 cells were analyzed for each experimental condition from 3 to 5 animals per genotype. The nuclei were labeled with DAPI (blue). Data are shown as mean ± SD; differences were not statistically significant using the one-way ANOVA and Bonferroni *post-hoc* test. Statistically significant differences are highlighted in red.

Since S0859 exhibited unrelated to its function as NBC family blocker effects on GABA_A_R transmission (Supplementary Figure 2) and taken into consideration that the NBCe1 family member is an interaction partner of KCC2, we investigated the putative relative contribution of NBCe1 in regulating KCC2 stimulus-induced changes of phosphorylation state in immature and differentiated neurons from mice with constitutive knock-out of NBCe1. Unfortunately, the *Nbce1*-deficient mice die at early postnatal stages (Gawenis et al., 2007). To bypass this problem, we studied the primary cultures of hippocampal neurons dissociated from 17-day embryos of wildtype (WT) and *Nbce1*^−/−^ (KO) littermates. The cultures were treated at either DIV4 with 10 μM staurosporine, a broad kinase inhibitor (Figure 3) or at DIV12 with 100 μM 4AP, a blocker of certain potassium channels and a model for epilepsy *in vitro* (Gonzalez-Sulser et al., 2011; Figure 4; Supplementary Figure 3), and subsequently, the expression of KCC2 whole protein, as well as its phosphorylation at S940 and T1007 were determined by immunofluorescence.

In immature DIV4 neurons, as shown in Figures 3A, B, the ratio of pKCC2 S940/total KCC2 was significantly downregulated following the exposure to staurosporine in WT but not in KO, compared to control cells (Supplementary Table S2). Moreover, in untreated neurons, the ratio of pKCC2 S940/total KCC2 was significantly reduced in KO, compared to WT littermates. Notably, as shown in Figures 3A, C, following staurosporine treatment, the ratio of pKCC2 T1007/total KCC2 was significantly downregulated in WT but not in KO, compared to the respective untreated cells (Supplementary Table S3), while the ratio of pKCC2 T1007/total KCC2 was similar between untreated cells of WT and KO (Supplementary Table S3).

Thus, these results indicate that in the absence of the NBCe1, the phosphorylation level of KCC2 and its sensitivity to staurosporine are different as compared to naïve conditions.

In DIV12 hippocampal neurons, as shown in Figures 4A, B, at baseline, the ratio of pKCC2 S940/total KCC2 was upregulated in KO, compared to WT. Moreover, the ratio of pKCC2 S940/total KCC2 was downregulated in 4AP-treated cells, compared to untreated controls in WT and KO (Supplementary Table S4). Similarly, as shown in Figures 4A, C, the baseline ratio of pKCC2 T1007/total KCC2 was significantly increased in KO compared to WT. In addition, 4AP treatment had the opposite effect on the ratio of pKCC2 T1007/total KCC2 in WT and KO. 4AP treatment significantly reduced the ratio of pKCC2 T1007/total KCC2 in WT, whereas it was significantly upregulated in KO (Supplementary Table S5).

In differentiated neurons, KCC2 reveals both intracellular and plasma membrane localization. Therefore, we next tested whether exposure to 4AP affected the trafficking of KCC2 to the plasma membrane in WT and KO neurons (Figure 4D). Representative line scans for KCC2 from control and 4AP-treated WT and KO neurons showed peaks of immunofluorescence in the periphery of the cells (arrows), presumably representing plasma membrane labeling, and no apparent differences were observed between experimental groups or genotypes. Counting of the neurons with membrane KCC2 confirmed this observation (Figure 4D; Supplementary Table S6).

Taken together, the results suggested an altered stimulus-induced phosphorylation pattern of KCC2 in the absence of NBCe1 with putative subsequent consequences on KCC2 activity.

### 3.4. Reversal potential of GABA_A_ responses in *Nbce1-*deficient neurons

Putative functional differences between WT and *Nbce1*-deficient neurons were determined. As a first test of the putative functional outcome of the interaction between KCC2 and NBCe1, we compared E_GABA_ in DIV12 WT and KO neurons that are dependent on [Cl^−^]_i_ and controlled by KCC2 (Medina et al., 2014; Moore et al., 2017). The E_GABA_ was assayed using gramicidin perforated patch-clamp recording that allows preserving the intracellular milieu (Friedel et al., 2015; Dumon et al., 2018).

**Figure 5:**
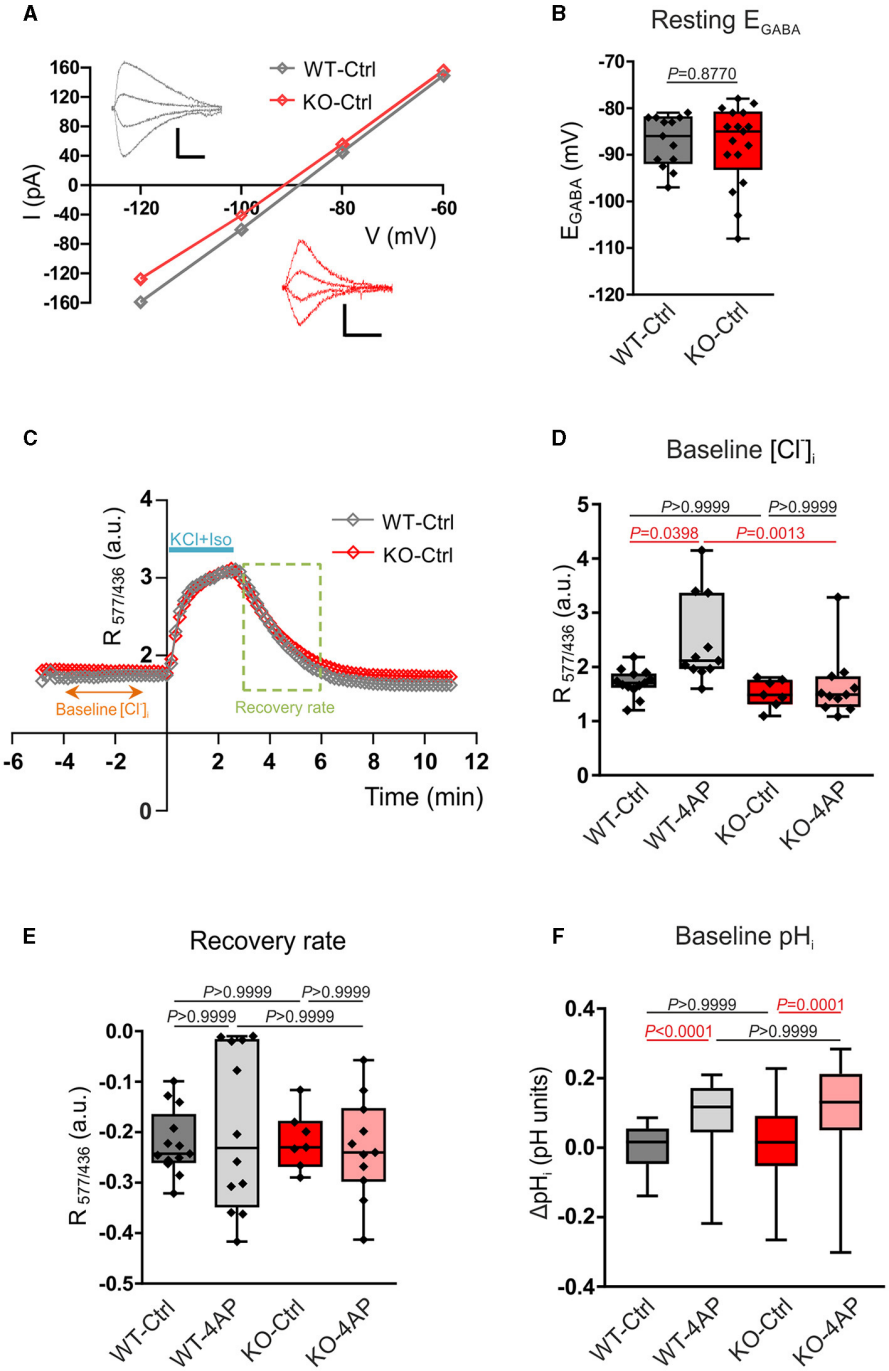
Assay of Cl^−^- and pH-related homeostasis in 12–13 DIV cultured hippocampal neurons of WT and *Nbce1* deficient (KO) mice. **(A)** Current (I)-Voltage (V) relationships for isoguvacine currents in WT and KO hippocampal (13 DIV) cultures. Gramicidin perforated patch clamp recordings. Insets depict the isoguvacine currents. Scale bars: 500 ms, 100 pA. **(B)** Box plots of E_GABA_ in the indicated conditions. The difference between WT and KO data was not significant (*p* = 0.88, Mann-Whitney test). **(C)** Examples of the dynamics of the ratio of F577/F436 fluorescence (R_577/436_) at resting conditions, during and after application of a solution containing 50 mM KCl and 30 μM isoguvacine (KCl + Iso). The recovery rate was quantified as the difference between R_577/436_ values recorded 180 and 10 s after the beginning of the washout of KCl and isoguvacine (period indicated using dashed square). **(D)** Population data of the R_577/436_ at resting conditions (baseline) calculated as the mean value of R_577/436_ in the somatodendritic region of the neuron during the time period from −4 to −1 min indicated in the plot C. **(E)** The box plots of the recovery rate of R_577/436_ after neuron overload with Cl^−^ illustrated in **(C)**. **(F)** Intracellular pH changes (ΔpH_i_) at resting condition (baseline) in WT and KO 12–13 DIV hippocampal neurons in controls (Ctrl) and after exposure to 100 μM 4AP. Cells were loaded with BCECF-AM dye and the changes in [H^+^]_i_ were monitored using the ratio F_440/488_ and converted into pH. ΔpH_i_ was significantly increased following exposure to 4AP in both WT and KO. The statistical analysis in **(D–F)** was made using the Kruskal-Wallis and Dunn *post-hoc* test. **(B, D, E)** The *n* was 8–15 neurons per condition (4 cultures). Individual values are indicated as filled diamonds. **(F)** The total number of cells for each experimental condition was 36–53 (4 cultures). Statistically significant differences are highlighted in red.

In our experimental design, the routine application of 10 μM bumetanide was implemented across all cultures to inhibit NKCC1 activity. This step was crucial, as NKCC1, like KCC2, relies on phosphorylation and could potentially contribute to phosphorylation-driven alterations in neuronal chloride balance (Inoue et al., 2012; Friedel et al., 2015; Watanabe and Fukuda, 2015). An analysis of recordings from both WT and KO neurons revealed comparable E_GABA_ values, displaying no statistically significant differences. Specifically, the median E_GABA_ values were −86 and −85 mV for WT and KO neurons, respectively (as depicted in Figures 5A, B; Supplementary Table S7). It is worth noting that these values concurred with E_GABA_ measurements recorded with bumetanide in cultured rat hippocampal neurons −83 mV (Friedel et al., 2015), −99 mV (Dumon et al., 2018), and −106.7 mV (Kontou et al., 2021).

Since Cl^−^ is the major carrier of GABA_A_R-mediated current, the obtained results indicate that the intracellular/extracellular gradients of Cl^−^ ions and the KCC2 activities are similar, respectively, in WT and KO neurons.

### 3.5. [Cl^−^]_i_ estimation using Cl^−^ sensitive genetically encoded probe

The above conclusion was further verified by measuring Cl^−^ -sensitive fluorescence using a ratiometric probe Clo-pHensorN (Arosio et al., 2010; Raimondo et al., 2013). The cultured WT and KO neurons were transfected with cDNA encoding Clo-pHensorN at DIV7, and measurements were taken on DIV13. Under resting conditions, both WT and KO showed similar values of R_577/436_ (Figures 5C, D; Supplementary Table S8), measured as described in the methods. These values were proportional to the [Cl^−^]_i_ and suggested the equality of resting [Cl^−^]_i_ in naïve WT and KO mice hippocampal cultures and were consistent with the equity of E_GABA_ in WT and KO described above. The 1 h treatment of cultures with 4AP, which strongly enhanced the ongoing neuronal activity (Supplementary Figure 3), provoked a significant shift of the resting R_577/436_ toward higher values (Figure 5D; Supplementary Table S8).

The resting level of [Cl^−^]_i_ in neurons depends, in addition to KCC2, on a large number of parameters that might compensate for the minor changes in KCC2 activity (Mahadevan and Woodin, 2016). We, therefore, produced an overload of neurons with Cl^−^ by applying in 2 min a solution containing 30 μM of isoguvacine and 50 mM of KCl in order to open Cl^−^ permeable GABA_A_R and create a strong inwardly directed Cl^−^ influx to depolarized neurons. The Cl^−^ overload could be observed on R_577/436_ traces illustrated in Figure 5C. After arresting the application of isoguvacine and KCl, the R_577/436_ values achieved the steady-state level and started to progressively decline to their initial values. The kinetics of the R_577/436_ decline reflected the Cl^−^ extrusion from the neuron that is, presumably, KCC2-dependent (Friedel et al., 2013; Hartmann et al., 2017).

Although the kinetics of the R_577/436_ recovery after overload were statistically non-different for all four studied conditions (Figure 5E; Supplementary Table S9), it is worth mentioning the higher variability of results in cultures treated with 4-AP (both in WT and KO). This clearly indicated a strong pressure of the treatment on the functioning of the entire complex of channels and transporters maintaining Cl^−^ homeostasis and highlights an important question for future studies.

### 3.6. Baseline pH_i_ estimation using proton-sensitive BCECF-AM

KCC2 is a putative neuronal ${\text{NH}}_{4}^{+}$ uptake pathway (Titz et al., 2006), another study has shown that several KCCs, among them KCC2, might be affected by changes in intracellular pH (Bergeron et al., 2003). We, therefore, additionally examined changes in intracellular [H^+^] at baseline in primary DIV12-13 hippocampal neurons from WT and KO. Cells were loaded with the proton-sensitive dye BCECF-AM, and the changes in [H^+^]_i_ were monitored ratiometrically. The ratio F_440/488_ was converted into pH, and the pH changes (ΔpH) between the experimental conditions were calculated, as described in the Material and Methods section. As shown in Figure 5F, at baseline, ΔpH_i_ was comparable between untreated WT and *Nbce1* deficient hippocampal neurons (Supplementary Table S10). Following treatment with 4AP, however, ΔpH_i_ at baseline was significantly increased compared to the untreated controls in both WT and *Nbce1*-deficient neurons.

## 4. Discussion

Cl^−^ and ${\text{HCO}}_{3}^{-}$ homeostasis are crucial parameters that control neuronal excitation and inhibition and therefore implicated in overall brain function in physiological and pathophysiological conditions. The neuronal KCC2 is the master regulator of [Cl^−^]_i_ in mature neurons, whereas pH_i_ is regulated by members of the *SLC4* family of bicarbonate transporters. The main findings of the present study are the following: (1) The study offers experimental proof of the protein-protein interaction between KCC2 and NBCe1. (2) The study illustrates that the KCC2/NBCe1 interaction is, presumably, involved in the mechanism of phosphorylation/dephosphorylation of the KCC2 at least at the residues S940 and T1007.

A recent interactome study has identified three members of the *SLC4* family, namely *SLC4A4* (NBCe1), *SLC4A8* (NDCBE), and *SLC4A10* (NCBE), as putative interaction partners of KCC2. Both SLC4A8 and SLC4A10 mediate Na^+^-driven chloride/bicarbonate exchange and are involved in neuronal intracellular pH regulation. SLC4A10 exhibits broad distribution and overlapping and/or complementary localization with KCC2 in distinct neuronal populations (Song et al., 2014), and mice lacking *Slc4a10* show reduced neuronal excitability (Jacobs et al., 2008). SLC4A8, which reveals a presynaptic distribution and is a key regulator of presynaptic pH, modulates glutamate release in a pH-dependent manner, and mice lacking *Slc4a8* display increased seizure threshold (Sinning et al., 2011). In contrast, SLC4A4 is the major glial pH regulator, which makes the observation of a putative KCC2/SLC4A4 interaction intriguing because the physiological function of NBCe1 in neurons is largely unknown. In the present study, we hypothesized that SLC4A4/KCC2 interaction in neurons is relevant under pathophysiological stimuli. This hypothesis was based on the following observations: (1) NBCe1 is expressed not only in astrocytes but also in neurons. (2) Although NBCe1 is apparently not a main molecular player in the regulation of intracellular neuronal pH under physiological conditions, the few studies on neurons that are available so far, indicate a putative function of neuronal NBCe1 under pathophysiological conditions, such as sustained membrane depolarization (Svichar et al., 2011), extracellular acid-base disturbances (Oehlke et al., 2013), ischemia/reperfusion (Sohn et al., 2011), or seizure (Kang et al., 2002). (3) KCC2 represents a pathway for neuronal ${\text{NH}}_{4}^{+}$ uptake, potentially leading to intracellular acidification (Titz et al., 2006), and vice versa, another study has shown that KCC2 is regulated by intracellular pH (Bergeron et al., 2003). (4) KCC2 is regulated under pathophysiological conditions as well (Payne et al., 2003; Boulenguez et al., 2010; Kahle et al., 2014; Puskarjov et al., 2014; Merner et al., 2015). (5) The observation of Smalley et al. (2020) that SLC4A4 and KCC2 are interaction partners. In the present study, as a first step, we investigated the impact of NBCe1/KCC2 interaction on the stimulus-induced phosphorylation profile of KCC2 *in vitro*.

Our results show that NBCe1 and KCC2 indeed colocalize in immature and mature mouse hippocampal neurons and co-immunoprecipitate in the immature hippocampus and adult mouse brain (Figure 1), thus arguing for a putative functional coupling. Applying the knowledge of NBCe1 gained from astrocytes and epithelial cells (Roussa et al., 2004; Rickmann et al., 2007; reviewed by Parker and Boron, 2013), it is apparent that NBCe1 and KCC2 share characteristics and regulation modes. They are involved in ionic homeostasis, are transcriptionally and post-translationally regulated by growth factors such as TGF-β2 (Roussa et al., 2016; Khakipoor et al., 2017; Rigkou et al., 2022), and their transport activity is critically dependent on (de)phosphorylation, mediated by common signaling pathways, among them PKC and WNK1/SPAK (Yang et al., 2011; Hong et al., 2013).

### 4.1. Implications of KCC2/NBCe1 interaction on KCC2 phosphorylation profile

In the present study, we used DIV4 immature and DIV12-14 mature neurons and exposed them to known KCC2 regulators. Although KCC2 expression in immature neurons is low, several studies have convincingly demonstrated the staurosporine-dependent changes of KCC2 phosphorylation in HEK-293 cells transfected with KCC2^WT^, as well as in immature neurons (Khirug et al., 2005; Inoue et al., 2012). Interestingly, following several brain pathologies, among them neurodevelopmental disorders, trauma, or epilepsy, neurons apparently adopt an earlier immature-like state (Nabekura et al., 2002; Boulenguez et al., 2010; Moore et al., 2017). For mature DIV12-14 neurons, we used 4AP as a stimulus. This choice was made taking into consideration the following observations: First, globally, 4AP is considered a model for epilepsy *in vitro* and *in vivo*. Second, although KCC2 has a complex role in disease pathology (reviewed by Cherubini et al., 2022), according to the prevailing notion, the dysfunction of KCC2 leads to the rise of intracellular Cl^−^ impairing GABA_A_-mediated inhibition, thereby disrupting the excitatory/inhibitory balance leading the brain to be more susceptible to seizures. Third, previous studies from our laboratory have shown the 4AP-dependent NBCe1 upregulation of protein and functional expression in mouse cortical and hippocampal astrocytes *in vitro* (Khakipoor et al., 2017).

Our results also show that in the absence of NBCe1, the profile of KCC2 phosphorylation at baseline and following treatment with staurosporine or 4AP is modified (Figures 3, 4). In *Nbce1-*deficient immature hippocampal neurons, baseline phosphorylation of KCC2 at S940 was downregulated, compared to WT, and exposure to staurosporine failed to reduce pKCC2 S940 and pKCC2 T1007. In *Nbce1*-deficient cultured mature neurons, the baseline level of pKCC2 at S940 and T1007 was upregulated compared to WT. Moreover, exposure to 4AP had an opposite effect on WT and KO with regard to pKCC2 T1007; whereas 4AP significantly decreased pKCC2 T1007 in WT, pKCC2 T1007 further increased after the application of 4AP in the KO. (De)phosphorylation-dependent regulation of KCC2 transport activity is well established (for review, see Hartmann and Nothwang, 2022). Specifically, the impact of (de)phosphorylation at S940 and T1007 has been analyzed most in-depth. (De)phosphorylation at S940 and T1007 is developmentally regulated and results in opposite effects on KCC2 transport activity. WNK1/SPAK/OSR1 pathway phosphorylates T1007 (de Los Heros et al., 2014), and its dephosphorylation by a not yet identified phosphatase increases KCC2 activity (Friedel et al., 2015). In contrast, PKC-mediated S940 phosphorylation increases KCC2 surface expression and stability, accompanied by increased KCC2 transport activity (Lee et al., 2007). Along this line, knock-in mice with homozygous phosphomimetic KCC2 mutations T906E/T1007E die early postnatally (Pisella et al., 2019; Watanabe et al., 2019), and in mice with T906A/T1007A mutations mimicking dephosphorylated forms of T906/T1007, the onset of seizures is delayed, and the severity of seizures attenuated (Moore et al., 2018). In contrast, in mice harboring S940A dephosphorylated-like mutation in KCC2, the development and lethality of status epilepticus are accelerated (Silayeva et al., 2015).

Several additional regulatory phospho-sites have been identified, and their effect on KCC2 function has been documented (Hartmann and Nothwang, 2022); however, appropriate experimental tools to study these sites are missing to date. Therefore, we cannot exclude that the absence of NBCe1 may affect the (de)phosphorylation of other KCC2 residues as well. What could be the underlying mechanism of NBCe1-dependent altered KCC2 phosphorylation? The results of our immunoprecipitation approach and colocalization experiments suggest that NBCe1 and KCC2 are part of an immunocomplex. It remains to be evaluated using appropriate approaches how exactly this complex is organized and whether NBCe1/KCC2 is a direct protein-protein interaction or does it involve intermediate partners. Regardless of the exact organization of the immunocomplex, one might speculate that a loss of NBCe1/KCC2 interaction might lead to structural changes resulting in modified accessibility of phosphorylatable KCC2 residues to kinases/phosphatases In this context, it is important to note that according to the targeting strategy to generate the *Nbce1* KO, no NBCe1 protein expression occurs (Gawenis et al., 2007). An indirect effect would imply subtle NBCe1-dependent changes of pH_i_, thereby altering the activity of kinases and phosphatases responsible for KCC2 (de)phosphorylation at S940 and T1007. Overall, a modified KCC2 phosphorylation profile in the absence of NBCe1 argues for an altered KCC2 transport activity. Alternatively, modified KCC2 phosphorylation could have no impact on KCC2 transport activity because they may compensate each other but rather affect the interaction of KCC2 with other proteins.

### 4.2. Implications of KCC2/NBCe1 interaction on KCC2 transport activity

In our efforts to elucidate the functional consequences of NBC/KCC2 interaction, we first investigated the pharmacological inhibition of NBCs by S0859, a molecule known to inhibit several electroneutral and electrogenic NBC isoforms. We examined the effect of S0859 on the KCC2 activity in stably transfected HEK-293 cells (Figure 2). HEK^KCC2WT^ cells are an established model to explore the changes in KCC2 activity, and they endogenously express K_v_ channels (Jiang et al., 2002) that can be blocked by 4AP. Our results showed that at baseline, blocking of NBCs had no effect on KCC2 activity, whereas staurosporine and 4AP-induced increase of KCC2 activity was prevented.

The same approach in primary hippocampal neurons, however, revealed that S0859 led to the inhibition of spontaneous GABA_A_R-mediated currents (Supplementary Figure 2). Consequently, S0859 was not found suitable for NBC study in any brain structures as its application will definitely block the inhibitory neurotransmission and extensive release of glutamate, which in turn will affect the properties of all neuronal networks and all brain cell types ranging from neurons to different types of glial cells. This finding is of particular importance because S0859 is broadly used as an NBC inhibitor in several *in vitro* and *in vivo* settings.

It remains unclear what are the functional consequences of NBCe1-dependent enhancements of KCC2 phosphorylation at residues S940 and T1007 observed in the present study. Our experiments involving analysis of the surface expression of KCC2 (Figure 4D) and recordings of E_GABA_, [Cl^−^]_i_, and pH_i_ (Figure 5) did not reveal any significant difference in these parameters between WT and *Nbce1* deficient hippocampal neurons. In addition, our results did not reveal the difference between *Nbce1* KO and WT in the kinetic of the Cl^−^ extrusion after imposed Cl^−^ overload that reflects the activity of KCC2. As mentioned above, the S940 phosphorylation of KCC2 is associated with enhancement of KCC2's surface expression and, potentially, activation of KCC2, whereas phosphorylation of T1007 is associated with an opposite action—internalization of KCC2 and reduction of KCC2 activity (Medina et al., 2014; Moore et al., 2017). So far, no studies have been undertaken to analyze the effects of simultaneous hyperphosphorylation of S940 and T1007. Both phosphorylation sites are present in an intrinsically disordered region that is situated between α8 and α9 helices (Hartmann and Nothwang, 2022). Here, phosphorylation of specific sites induces conformational changes that affect KCC2 transport activity (Hartmann and Nothwang, 2022). Since the phosphorylation of S940 enhances, and the phosphorylation of T1007 reduces KCC2 activity, it is highly probable that structural changes of KCC2's C-terminus caused by phosphorylation of both sites compensate for each other.

Interestingly, in WT hippocampal neurons, treatment with 4AP significantly increased the baseline [Cl^−^]_i_ and ΔpH_i_, whereas in *Nbce1* deficient neurons ΔpH_i_, but not [Cl^−^]_i_, was significantly increased.

Neuronal [Cl^−^]_i_ is set through the coordinated action of several Cl^−^-extruding and Cl^−^-loading transport mechanisms as well as ion-transport independent mechanisms (reviewed by Doyon et al., 2016; Rahmati et al., 2018). The ion-transport mechanisms involve various channels and transporters, among which particular importance is attributed to KCC2, NKCC1, Cl^−^ channels (voltage-gated, Ca^2+^-activated, pH-sensitive), and several members of the *SLC4* family. The putative ion-transport independent mechanism involves negatively charged ions that are unable to cross the membrane (Donnan effect). Computational modeling aimed to elucidate the importance of these different components in controlling neuronal [Cl^−^]_i_ (Düsterwald et al., 2018) illustrated that changing the levels of impermeant anions had an impact on cell volume but did not substantially alter the chloride-driving force. In contrast, modulating the activity of cation-chloride cotransporters, including KCC2, had a profound effect on the chloride driving force, suggesting that these transporters are crucial determinants of [Cl^−^]_i_ regulation.

Although KCC2 is the main Cl^−^-extruding mechanism in neurons, under the experimental conditions in this study, its effect might have been masked by the effect of other Cl^−^-extruding systems. Comparing the results obtained in HEK-293 cells (Figure 2) with those in DIV12 hippocampal neurons (Figure 4), it is clear that the effect of NBC inhibition on 4AP-dependent regulation of KCC2 transport activity was not reproducible in primary neurons. S0859 inhibits not only NBCe1 but also other NBC isoforms. Therefore, the lack of significant effect (i.e., downregulation of KCC2 activity in *Nbce1* KO) can be explained by either the effect in HEK cells was not attributed to NBCe1 at all or by compensatory upregulation of other NBCs in the *Nbce1* KO. Moreover, the lack of significance in KCC2 transport activity does not necessarily indicate that the function of the neuron has not changed; KCC2 may also function independently of Cl^−^ in a non-canonical way (Virtanen et al., 2021), resulting in morphological changes via interaction with other proteins.

A main limitation of our study is the investigation of NBCe1/KCC2 interactions in neuronal cultures, where glial cell development was intentionally reduced during the preparation process. The neuronal environment, including nutrient availability, extracellular organic molecules, and ion concentrations, is little dependent on glial cells. *In vivo*, the situation is much more complex, and in addition to NBCe1/KCC2 interaction in neurons, an astrocytic NBCe1/neuronal KCC2 may be a mode for direct or indirect glia-neuron communication. Since astrocytic NBCe1 regulates not only intracellular astrocytic pH but also extracellular pH, the regulation of astrocytic NBCe1 might influence neuronal properties independently of an NBCe1/KCC2 interaction. One might even speculate that neuronal KCC2 may interact directly with neuronal and/or astrocytic NBCe1, thereby modulating its phosphorylation profile and/or serving as a mode of crosstalk between glia and neurons. To test such biological significance, additional studies are needed.

In conclusion, the present study shows the protein-protein interaction between KCC2 and NBCe1 that is involved in the least regulation of KCC2 protein phosphorylation. This finding opens a perspective for future *in vitro* and *in vivo* studies aimed at a deeper understanding of the functional importance of this interaction.

## Data availability statement

The original contributions presented in the study are included in the article/Supplementary material, further inquiries can be directed to the corresponding author.

## Ethics statement

Ethical approval was not required for the studies on humans in accordance with the local legislation and institutional requirements because only commercially available established cell lines were used. The animal study was approved by the Institutional Animal Care and Use Committee of the University of Freiburg and the City of Freiburg (Authorizations: X19/09C, X22/11B, and G-21-140). The study was conducted in accordance with the local legislation and institutional requirements.

## Author contributions

AP: Formal analysis, Investigation, Methodology, Writing—review and editing. MH: Formal analysis, Investigation, Methodology, Writing—review and editing. MG: Formal analysis, Investigation, Methodology, Writing—review and editing. BH: Formal analysis, Investigation, Methodology, Writing—review and editing. A-MH: Formal analysis, Investigation, Methodology, Writing—review and editing. IM: Conceptualization, Formal analysis, Investigation, Methodology, Supervision, Writing—original draft, Writing—review and editing. ER: Conceptualization, Formal analysis, Investigation, Methodology, Project administration, Supervision, Writing—original draft, Writing—review and editing.
