# Supplementary material for: K+/Cl− cotransporter 2 (KCC2) and Na+/HCO3− cotransporter 1 (NBCe1) interaction modulates profile of KCC2 phosphorylation
Source: Front Cell Neurosci. 2023 Oct 10;17:1253424. doi: 10.3389/fncel.2023.1253424 (PMC10595033; doi:10.3389/fncel.2023.1253424)
Supplement: Supplementary file 1 [file Data_Sheet_1.PDF]

Supplementary Material

K^+/Cl^- cotransporter 2 (KCC2) / Na^+/HCO_3^- cotransporter 1 (NBCe1) interaction modulates profile of KCC2 phosphorylation.

Abhishek Pethe[1], Mira Hamze[3], Marina Giannaki[1], Bernd Heimrich[2], Igor Medina[3], Anna-Maria Hartmann[4,5], Eleni Roussa[1*]

*** Correspondence:** Dr. Eleni Roussa: eleni.roussa@anat.uni-freiburg.de

Supplementary Method

Preparation of organotypic slice cultures

Hippocampal slice cultures (HSC) were prepared from P2-P4 day-old wildtype or *Nbce1* deficient mice. Briefly, brains were aseptically removed from the skulls and transferred into culture dishes containing cold preparation buffer solution. Under binocular observation, hippocampi were dissected and cut into 400 µm slice perpendicular to the longitudinal axis by means of a tissue chopper. The slices were placed on translucent porous membranes and transferred into six-well plates filled with 1.2 ml medium supplemented with 2 mM glutamine at pH 7.3. HSC were maintained at 37°C in an incubator in an atmosphere of humidified air and 5 % CO_2. Hippocampal slices were cultivated under standard conditions for 35 days with medium change 3 times weekly (for details see Novotny et al., 2016). Subsequently, slice cultures were processed for protein isolation and western blot analysis.

1 Supplementary Figures and Tables

1.1 Supplementary Figures

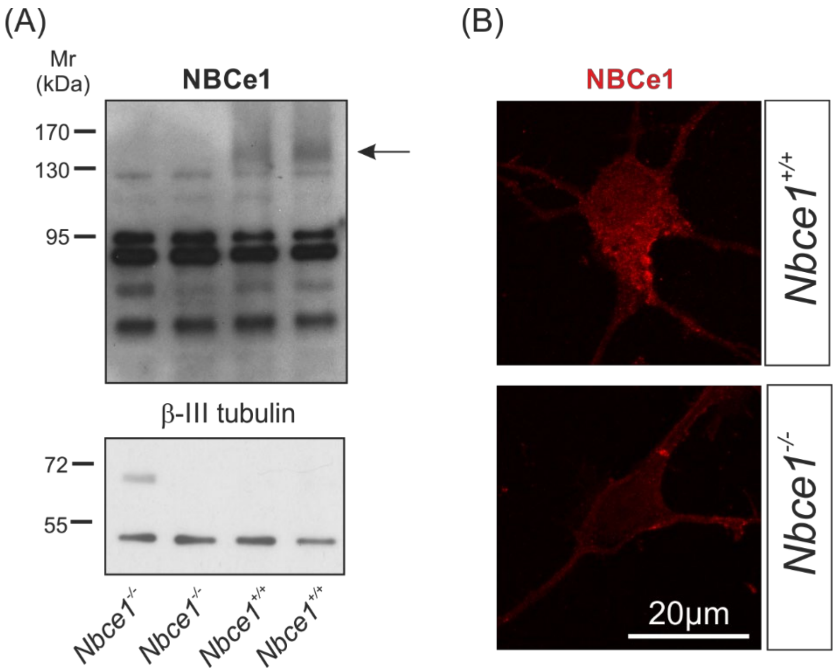

Supplementary Figure 1. Specificity of NBCe1 antibody. (A) Immunoblot analysis using NBCe1 antibody in organotypic mouse hippocampal slices cultured for 35 days in vitro derived from *Nbce1*$^{+/+}$ (lanes 3 and 4) and *Nbce1*$^{-/-}$ (lanes 1 and 2) mouse pups. The ~130-140kDa immunoreactive band was abolished in the knockout samples. (B) Immunofluorescence confocal microscopy for NBCe1 (in red) on mouse primary immature hippocampal neurons at day *in vitro* 4 derived from *Nbce1*$^{+/+}$ and *Nbce1*$^{-/-}$ mice. NBCe1 immunolabeling was greatly depleted in the knockout neurons.

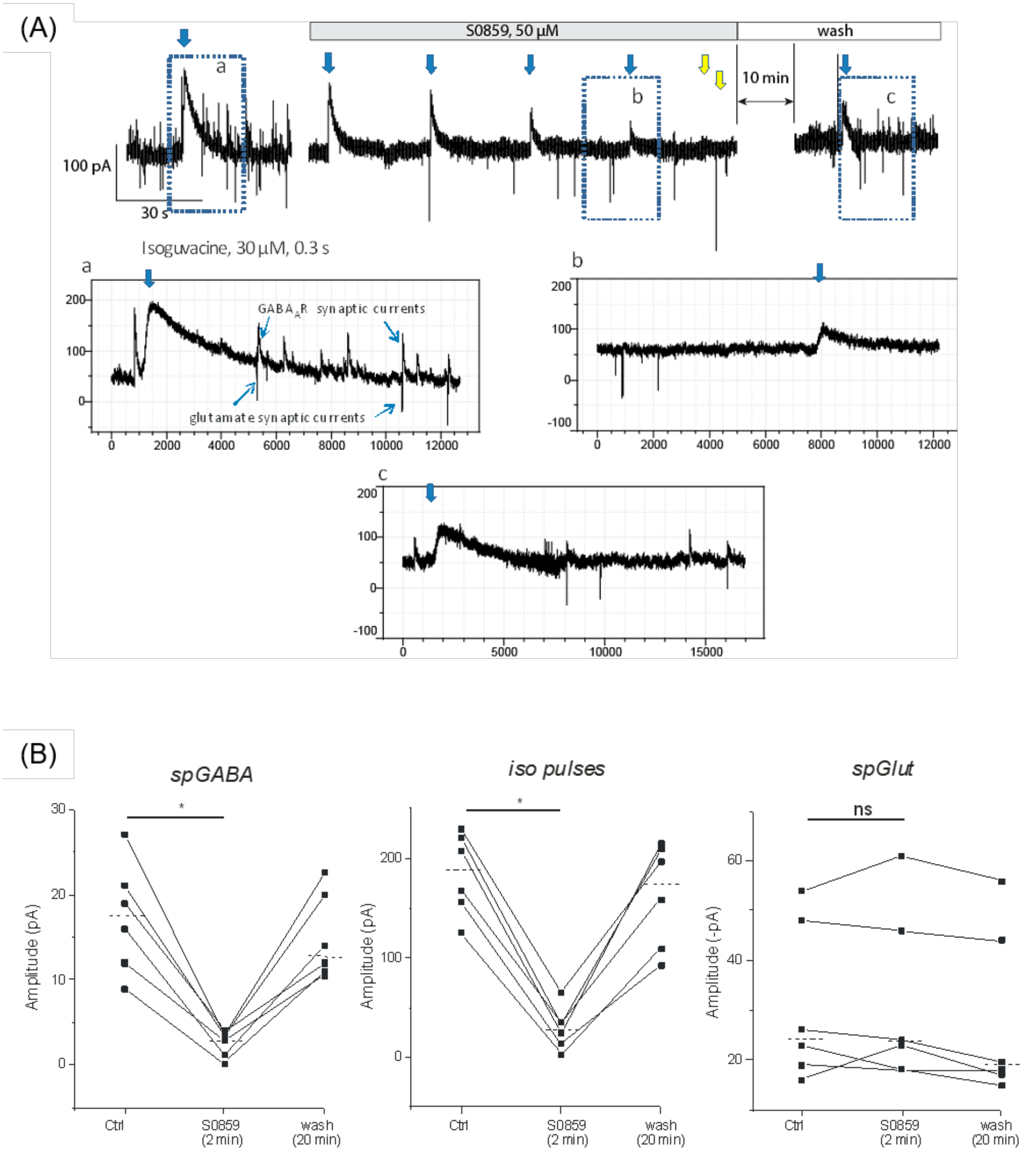

Supplementary Figure 2. Application of 50 μM S0859 to DIV12-13 cultured hippocampal neurons produced a strong and reversible inhibition of GABAergic, but not glutamatergic responses. (A) Representative trace of the long-lasting whole-cell recording (upper trace) of ion current in voltage clamp mode. The insets illustrate the selected fragments with higher time resolution. During recording, brief jets (300 ms) of external solution containing 30 μM isoguvacine were applied locally to the recorded neuron (indicated using arrows). The patch recording electrodes (4 to 7 megaohms) were filled with a solution containing 100 mM K-gluconate, 10 mM KCl, 10 mM Hepes, 1.1 mM EGTA,

0.1 mM $CaCl_2$, 4 mM Mg–adenosine 5′-triphosphate, and 0.3 mM Na–guanosine 5′-triphosphate. The pH of the intracellular solution was adjusted to 7.2, and the osmolality was adjusted to 280 mOsmol liter^{-1}. The access resistance ranged between 15 and 30 megaohms. With this recording solution, that contained 10 mM of Cl^-, the spontaneous $GABA_A$R-mediated postsynaptic currents (spGABA) reversed at −70 mV. spGABA and spGlut (spontaneous glutamate receptors-mediated postsynaptic currents) were recorded at a holding potential of −45 mV. At this potential, spGABA are outward and Glut-PSCs are inward. Notice the complete disappearance of spGABA events and strong decrease of response to isoguvacine 2 min after S0859 application (inset b). The responses to isoguvacine further decreased 30 s later (yellow arrows in upper trace). (B) Amplitudes of spGABA events, responses to isoguvacine applications and spGlut events measured before, 2 minutes after beginning of S0859 application and 20 minutes after wash-out of S0859. *, $p<0.05$ (Paired sample Wilcoxon Signed Rank test). The median values are shown using horizontal dashed lines.

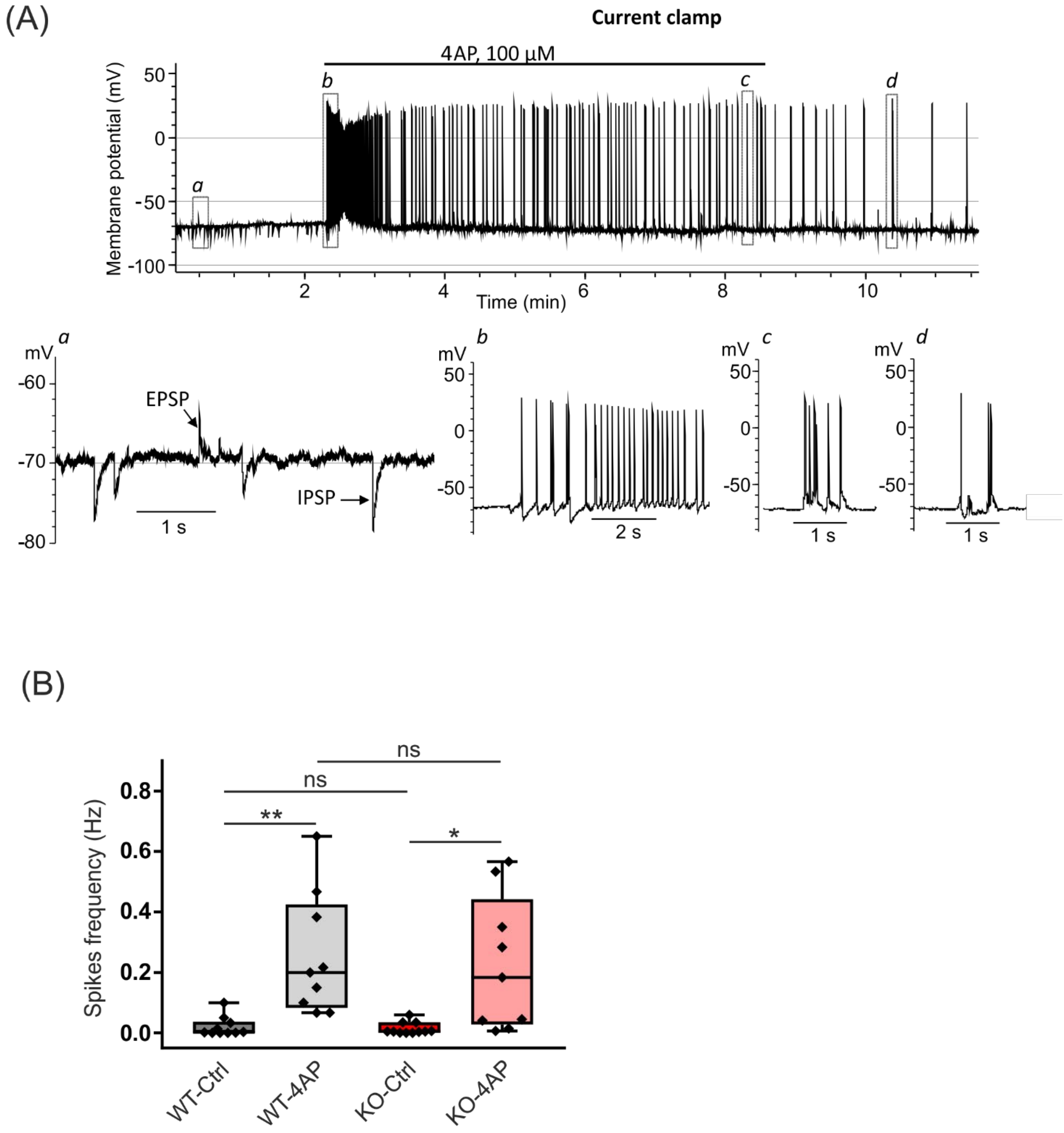

Supplementary Figure 3. Application of 4-aminopyridine (4AP) to DIV12-13 cultured hippocampal neurons produced a strong increase of the ongoing neuronal activity. (A) Representative trace of the long-lasting recording (upper trace) of membrane potential from *Nbce1* deficient hippocampal neuron in current clamp mode. Gramicidin-perforated patch. Bottom plots illustrate fragments of the recording at faster time scale and indicated using letters *a, b, c* and *d* above upper trace. Notice the absence of the spikes during resting period before application of the 4AP. The recorded neuronal activity during resting period (see inset *a*) is represented by spontaneous depolarizing EPSPs (excitatory postsynaptic potentials) and hyperpolarizing IPSPs (inhibitory postsynaptic potentials).The application of 4AP (100 µM) produced instant high frequency firing of the neuronal network (inset *b*) whose frequency

decreased within the time (inset *c*). After washing of the 4AP the spontaneous spiking activity decreased, but did not disappeared (inset *d*) indicating on the long lasting 4AP dependent changes in the cultured neurons network properties. (B) Overall frequency of the neuron spiking of control WT and *Nbce1* deficient neuronal cultures at resting conditions and after 60 min of incubation with 4AP.

1.2 Supplementary Tables

Table S1: Statistical details and comparisons among samples illustrated in Fig. 2. Purple highlighting indicates cases when difference is statistically significant at 0.05 level ($p<0.05$).

Tl^+ uptake normalized to Ctrl (KCC2) in HEK-293 cells (in %)						
**Condition**	**Median**	**Mean**	**Std. Deviation**	**Statistical Test**	**Comparisons**	***p* Value (adjusted for multiplicity)**
Ctrl (Mock)	30.50	38.65	23.42	One way ANOVA with Bonferroni's post hoc		
Ctrl + S0859 (Mock)	42.86	41.57	9.00			
Stauro (Mock)	48.39	50.65	17.02			
Stauro + S0859 (Mock)	43.55	47.79	13.65			
4AP (Mock)	14.91	21.98	17.43			
4AP + S0859 (Mock)	26.09	29.35	10.42		Ctrl (KCC2) vs Ctrl + S0859 (KCC2)	0.7253
Ctrl (KCC2)	101.5	100.00	10.67		Ctrl (KCC2) vs Stauro (KCC2)	<0.0001
Ctrl + S0859 (KCC2)	97.40	90.69	15.09		Ctrl + S0859 (KCC2) vs Stauro + S0859 (KCC2)	>0.9999
Stauro (KCC2)	131.60	137.60	18.87		Stauro (KCC2) vs Stauro + S0859 (KCC2)	<0.0001
Stauro + S0859 (KCC2)	81.82	84.12	12.48		Ctrl (KCC2) vs 4AP (KCC2)	0.0061
4AP (KCC2)	115.40	117.10	19.98		Ctrl + S0859 (KCC2) vs 4AP + S0859 (KCC2)	0.0436
4AP + S0859 (KCC2)	75.00	74.55	10.68		4AP (KCC2) vs 4AP + S0859 (KCC2)	<0.0001

Table S2: Statistical details and comparisons among samples illustrated in Fig. 3B. Purple highlighting indicates cases when difference is statistically significant at 0.05 level (*p*<0.05).

pKCC2 S940 / Total KCC2						
**Condition**	**Median**	**Mean**	**Std. Deviation**	**Statistical Test**	**Comparisons**	***p* Value (adjusted for multiplicity)**
WT Ctrl	1.13	1.29	0.57	Kruskal-Wallis and Dunn's post hoc test	WT Ctrl vs WT Stauro	0.0140
WT Stauro	1.04	1.05	0.39		WT Ctrl vs KO Ctrl	0.0001
KO Ctrl	0.89	1.01	0.46		WT Stauro vs KO Stauro	0.0356
KO Stauro	0.78	1.02	0.63		KO Ctrl vs KO Stauro	0.9596

Table S3: Statistical details and comparisons among samples illustrated in Fig. 3C. Purple highlighting indicates cases when difference is statistically significant at 0.05 level (*p*<0.05).

pKCC2 T1007 / Total KCC2						
**Condition**	**Median**	**Mean**	**Std. Deviation**	**Statistical Test**	**Comparisons**	***p* Value (adjusted for multiplicity)**
WT Ctrl	1.26	1.26	0.66	Kruskal Wallis and Dunn's post hoc test	WT Ctrl vs WT Stauro	0.0022
WT Stauro	0.84	0.89	0.35		WT Ctrl vs KO Ctrl	0.3732
KO Ctrl	0.92	1.06	0.52		WT Stauro vs KO Stauro	>0.9999
KO Stauro	0.84	0.95	0.40		KO Ctrl vs KO Stauro	>0.9999

Table S4: Statistical details and comparisons among samples illustrated in Fig. 4B. Purple highlighting indicates cases when difference is statistically significant at 0.05 level (*p*<0.05).

pKCC2 S940 / Total KCC2						
**Condition**	**Median**	**Mean**	**Std. Deviation**	**Statistical Test**	**Comparisons**	***p* Value (adjusted for multiplicity)**
WT Ctrl	1.57	1.79	0.89	Kruskal Wallis and Dunn's post hoc test	WT Ctrl vs WT 4AP	0.0006
WT 4AP	1.36	1.39	0.68		WT Ctrl vs KO Ctrl	<0.0001
KO Ctrl	2.07	2.08	0.53		WT 4AP vs KO 4AP	0.0001
KO 4AP	1.57	1.78	0.73		KO Ctrl vs KO 4AP	<0.0001

Table S5: Statistical details and comparisons among samples illustrated in Fig. 4C. Purple highlighting indicates cases when difference is statistically significant at 0.05 level ($p<0.05$).

pKCC2 T1007 / Total KCC2						
**Condition**	**Median**	**Mean**	**Std. Deviation**	**Statistical Test**	**Comparisons**	***p* Value (adjusted for multiplicity)**
WT Ctrl	0.65	0.73	0.33	Kruskal Wallis and Dunn's post hoc test	WT Ctrl vs WT 4AP	0.0003
WT 4AP	0.49	0.54	0.26		WT Ctrl vs KO Ctrl	0.0006
KO Ctrl	0.82	0.95	0.46		WT 4AP vs KO 4AP	<0.0001
KO 4AP	1.07	1.14	0.47		KO Ctrl vs KO 4AP	0.0014

Table S6: Statistical details and comparisons among samples illustrated in Fig. 4D.

KCC2 Line Scans (in %)						
**Condition**	**Median**	**Mean**	**Std. Deviation**	**Statistical Test**	**Comparisons**	***p* Value (adjusted for multiplicity)**
WT Ctrl	73.68	73.13	4.31	One way ANOVA with Bonferroni's post hoc	WT Ctrl vs WT 4AP	>0.9999
WT 4AP	75.00	77.02	6.82		WT Ctrl vs KO Ctrl	>0.9999
KO Ctrl	76.19	76.81	3.85		WT 4AP vs KO 4AP	>0.9999
KO 4AP	81.25	77.59	11.40		KO Ctrl vs KO 4AP	>0.9999

Table S7: Statistical details and comparisons among samples illustrated in Fig. 5B

Resting E_{GABA}						
**Condition**	**Median**	**Mean**	**Std. Deviation**	**Statistical Test**	**Comparisons**	***p* Value**
WT Ctrl	-86	-87.12	5.45	Mann-Whitney test	WT Ctrl vs KO Ctrl	0.88
KO Ctrl	-85	-88.00	8.65			

Table S8: Statistical details and comparisons among samples illustrated in Fig. 5D. Purple highlighting indicates cases when difference is statistically significant at 0.05 level (p<0.05).

Baseline $[Cl^-]_i$						
**Condition**	**Median**	**Mean**	**Std. Deviation**	**Statistical Test**	**Comparisons**	***p* Value (adjusted for multiplicity)**
WT Ctrl	1.703	1.71	0.25	Kruskal Wallis and Dunn's post hoc test	WT Ctrl vs WT 4AP	0.0398
WT 4AP	2.113	2.459	0.80		WT Ctrl vs KO Ctrl	>0.9999
KO Ctrl	1.484	1.514	0.26		WT 4AP vs KO 4AP	0.0013
KO 4AP	1.489	1.643	0.60		KO Ctrl vs KO 4AP	>0.9999

Table S9: Statistical details and comparisons among samples illustrated in Fig. 5E.

Recovery rate						
**Condition**	**Median**	**Mean**	**Std. Deviation**	**Statistical Test**	**Comparisons**	***p* Value (adjusted for multiplicity)**
WT Ctrl	-0.243	-0.221	0.06	Kruskal Wallis and Dunn's post hoc test	WT Ctrl vs WT 4AP	>0.9999
WT 4AP	-0.232	-0.196	0.16		WT Ctrl vs KO Ctrl	>0.9999
KO Ctrl	-0.230	-0.217	0.06		WT 4AP vs KO 4AP	>0.9999
KO 4AP	-0.240	-0.232	0.10		KO Ctrl vs KO 4AP	>0.9999

Table S10: Statistical details and comparisons among samples illustrated in Fig. 5F. Purple highlighting indicates cases when difference is statistically significant at 0.05 level (p<0.05).

Baseline ΔpH_i (pH units)						
**Condition**	**Median**	**Mean**	**Std. Deviation**	**Statistical Test**	**Comparisons**	***p* Value (adjusted for multiplicity)**
WT Ctrl	0.0164	2.597e-006	0.07	Kruskal Wallis and Dunn's post hoc test	WT Ctrl vs WT 4AP	<0.0001
WT 4AP	0.1174	0.094	0.10		WT Ctrl vs KO Ctrl	>0.9999
KO Ctrl	0.0154	0.017	0.12		WT 4AP vs KO 4AP	>0.9999
KO 4AP	0.1311	0.111	0.13		KO Ctrl vs KO 4AP	0.0001

Table S11: Statistical details and comparisons among samples illustrated in Supplementary Fig. 2B. Purple highlighting indicates cases when difference is statistically significant at 0.05 level (*p*<0.05).

Spikes frequency (Hz)						
**Condition**	**Median**	**Mean**	**Std. Deviation**	**Statistical Test**	**Comparisons**	***p* Value (adjusted for multiplicity)**
WT Ctrl	0.0025	0.0203	0.0327	Kruskal Wallis and Dunn's post hoc test	WT Ctrl vs WT 4AP	0.0050
WT 4AP	0.20	0.2556	0.2024		WT Ctrl vs KO Ctrl	>0.9999
KO Ctrl	0.005	0.0141	0.0199		WT 4AP vs KO 4AP	>0.9999
KO 4AP	0.1833	0.2250	0.2209		KO Ctrl vs KO 4AP	0.0184

2 Supplementary References:

Novotny, R., Langer, F., Mahler, J., Skodras, A., Vlachos, A., Wegenast-Braun, B.M., et al. (2016). Conversion of synthetic Aβ to in vivo active seeds and amyloid plaque formation in a hippocampal slice culture model. J. Neurosci. 36, 5084-5093. DOI: 10.1523/JNEUROSCI.0258-16.2016
